# Macrophages and β-cells are responsible for CXCR2-mediated neutrophil infiltration of the pancreas during autoimmune diabetes

**DOI:** 10.15252/emmm.201404144

**Published:** 2014-06-26

**Authors:** Julien Diana, Agnès Lehuen

**Affiliations:** 1 Institut National de la Santé et de la Recherche Médicale (INSERM), U1151, Necker-Enfants Malades Institute (INEM), Necker HospitalParis, France; 2 Sorbonne Paris Cité, Université Paris DescartesParis, France; 3 Institut National de la Santé et de la Recherche Médicale (INSERM), U1016, Cochin Institute, Cochin HospitalParis, France; 4 Laboratoire d'Excellence INFLAMEXParis, France

**Keywords:** autoimmunity, beta cell, diabetes, innate, neutrophil

## Abstract

Autoimmune type 1 diabetes (T1D) development results from the interaction between pancreatic β-cells, and the innate and the adaptive immune systems culminating with the destruction of the insulin-secreting β-cells by autoreactive T cells. This diabetogenic course starts during the first postnatal weeks by the infiltration of the pancreatic islets by innate immune cells and particularly neutrophils. Here, we aim to determine the cellular and molecular mechanism leading to the recruitment of this neutrophils in the pancreatic islets of non-obese diabetic (NOD) mice. Here, we show that neutrophil recruitment in the pancreatic islets is controlled by inflammatory macrophages and β-cells themselves. Macrophages and β-cells produce the chemokines CXCL1 and CXCL2, recruiting CXCR2-expressing neutrophils from the blood to the pancreatic islets. We further show that pancreatic macrophages secrete IL-1β-inducing CXCR2 ligand production by the β-cells. Finally, the blockade of neutrophil recruitment at early ages using CXCR2 antagonist dampens the diabetogenic T-cell response and the later development of autoimmune diabetes, supporting the therapeutic potential of this approach.

**Subject Categories** Immunology; Metabolism

## Introduction

Type 1 diabetes (T1D) is an autoimmune disorder engendered by the deleterious actions of several cell types from both the innate and the adaptive immune systems (Lehuen *et al*, 2010). We have recently revealed using the non-obese diabetic (NOD) mouse model that autoimmune diabetes is initiated in the pancreatic islets during the first postnatal weeks and requires the crosstalk between various innate immune cells including plasmacytoid dendritic cells (PDCs), B-1a cells, and neutrophils (Diana *et al*, 2013). We have shown that in neonatal NOD mice, neutrophils transiently infiltrate the pancreatic islets, produce the cathelicidin-related antimicrobial peptide (CRAMP), and participate with the B-1a cells to the stimulation of IFN-α-secreting pDCs in the pancreatic islets. This innate cell interplay is critical for diabetes development likely by the activation of conventional DCs (cDCs) and their migration to the lymph nodes where they present β-cell antigens to the autoreactive T cells (Atkinson *et al*, 2014). However, one important question remains: How neutrophils are recruited in the pancreatic islets of neonatal NOD mice? The infiltration of immune cells in the pancreatic islets during the T1D development has been extensively investigated revealing a role for CCR2 and CCR5 on myeloid cells and a role for CXCR3 and CCR5 on lymphoid cells (Eizirik *et al*, 2009). As neutrophils have been identified only recently in the pancreatic islets of neonatal NOD mice (Diana *et al*, 2013), the chemokines and chemokine receptor(s) implicated in their recruitment remained to determine. Various studies have demonstrated that neutrophil trafficking is a tightly regulated phenomenon from the release of neutrophils from the bone marrow (BM) into the circulation and then into the inflamed tissues (Sadik *et al*, 2011; Kolaczkowska & Kubes, 2013). The chemokines responsible for the recruitment of neutrophils in the tissue can be produced by both immune and non-immune cells such as macrophages and fibroblasts, respectively (Sadik *et al*, 2011). Various studies have shown that murine and human β-cells are able to produce various chemokines under inflammatory conditions participating to the recruitment of immune cells (Eizirik *et al*, 2009; Ortis *et al*, 2010; Sarkar *et al*, 2012). In this study, we hypothesized that β-cells may play a direct role in the recruitment of neutrophils in the pancreatic islets of neonatal NOD mice, leading to T1D development.

## Results

### Neutrophils infiltrate the pancreatic islets of neonatal NOD mice

**Figure 1:**
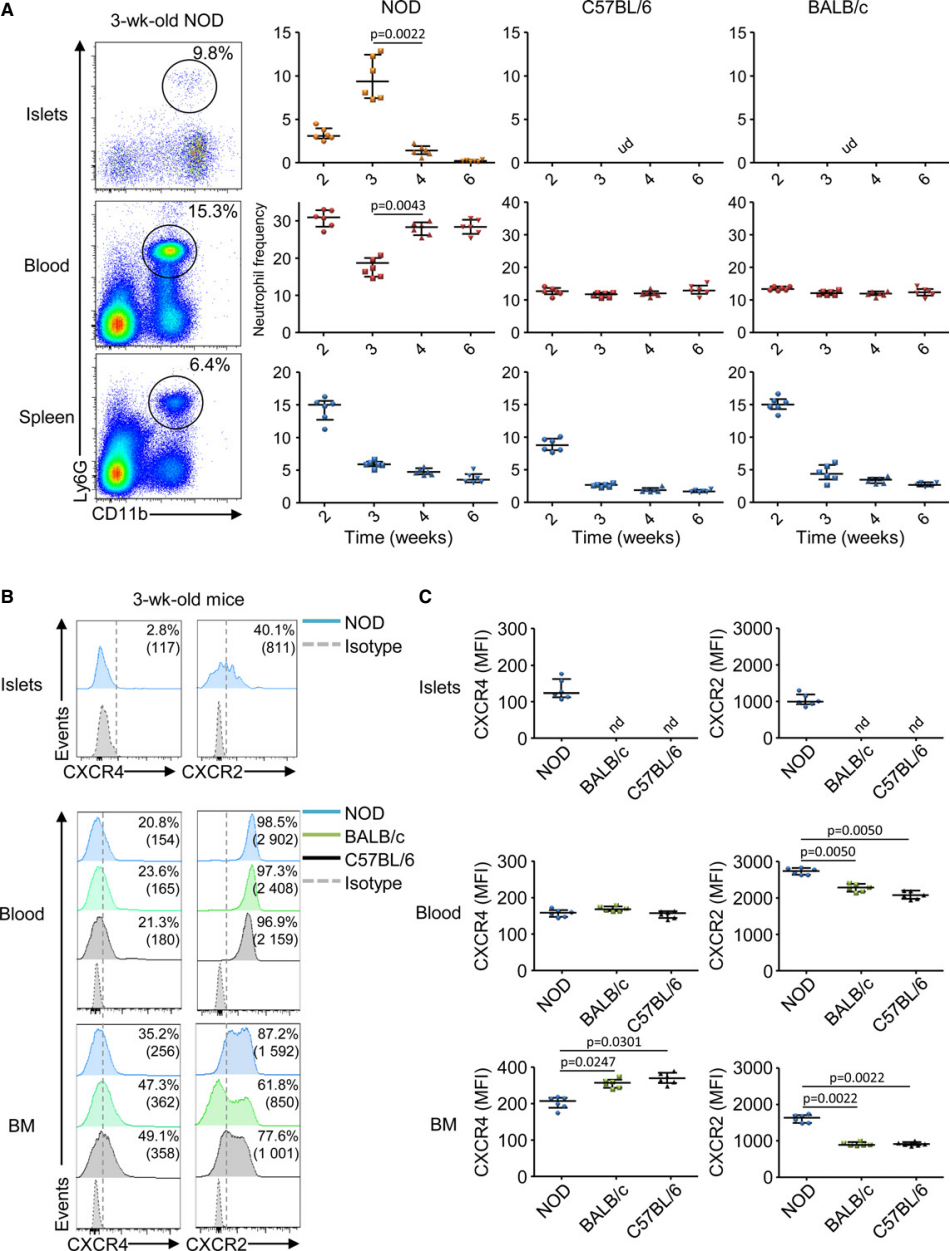
Neutrophil phenotype in neonatal NOD mice A   Kinetic analysis of neutrophils in the pancreatic islets, blood and spleen of female NOD, C57BL/6, and BALB/c mice. Cells were harvested at various age and stained for CD45, CD11b, and Ly6G expressions. Frequency of neutrophils among CD45^+^ cells is represented. Data are representative (flow cytometry dot plot) or are median ± interquartile range from six independent experiments with three pooled mice for each group. UD: undetectable. B, C   Analysis of CXCR4 and CXCR2 expressions on neutrophils in 3-week-old mice. Cells from the pancreatic islets, blood, and bone marrow (BM) were recovered from female NOD, C57BL/6, and BALB/c mice and stained for CD45, CD11b, Ly6G, CXCR4, and CXCR2 expressions. The frequency and mean fluorescence intensity (MFI) of CXCR4 and CXCR2 on neutrophils are represented. Data are representative (B) or are median ± interquartile range (C) from three independent experiments with two independent mice for each group. ND: not determined.

In this study, we have investigated the mechanism of recruitment of the neutrophils in the pancreatic islets of neonatal autoimmune NOD mice. Kinetic analysis revealed that neutrophils (CD45^+^ CD11b^+^ Ly6G^+^) infiltrated the pancreatic islets at 2 weeks of age and their frequency and absolute number increased until 3 weeks of age specifically in the NOD mice while neutrophils were undetectable in the islets of non-autoimmune C57BL/6 and BALB/c mice (Fig 1A and Supplementary Fig S1A). Strikingly, in the blood, a transient neutropenia was observed between 2 and 3 weeks of age in the NOD mice while neutrophil frequency and absolute number remained unaffected in the two non-autoimmune mice (Fig 1A and Supplementary Fig S1A). It is noteworthy that the frequency and absolute number of circulating neutrophils at any ages was higher in the NOD mice compared with the C57BL/6 and BALB/c mice (Fig 1A and Supplementary Fig S1A). Together, these data support that during the first postnatal weeks, some neutrophils were recruited to the pancreatic islets of the NOD mice and not in the pancreatic islets of the non-autoimmune mice.

### Circulating neutrophils harbor a mature phenotype in NOD mice

Neutrophil trafficking in mice is mainly regulated by two chemokine receptors: CXCR2 and CXCR4 (Eash *et al*, 2009, 2010; Sadik *et al*, 2011; Sanz & Kubes, 2012). High CXCR4 expression retains immature neutrophils in the BM while high CXCR2 expression and the presence of its ligands (mainly CXCL1 and CXCL2) allow the mobilization of mature neutrophils in the blood and their subsequent recruitment at inflamed sites. We observed that in the NOD mice, circulating neutrophils harbor a higher expression of CXCR2 and BM-neutrophils harbor a lower expression of CXCR4 as compared with their respective counterparts from non-autoimmune mice (Fig 1B and C). Accordingly, circulating NOD neutrophils harbored a more mature phenotype (CD11b^high^ and CD62L^low^) as compared with circulating neutrophils from non-autoimmune mice (Supplementary Fig S2). In the pancreatic islets, NOD neutrophils harbored a weak expression of CXCR4 and a mild expression of CXCR2 as expected for activated neutrophils (Tikhonov *et al*, 2001), and additionally, the exposure of the chemokine receptor to its ligands in the tissue would lead to receptor internalization and degradation (Baugher & Richmond, 2008). These data support that circulating NOD neutrophils harbored a mature phenotype likely favoring their recruitment from the blood to the inflamed islets.

### CXCR2 is required for the recruitment of neutrophils in the pancreatic islets

**Figure 2:**
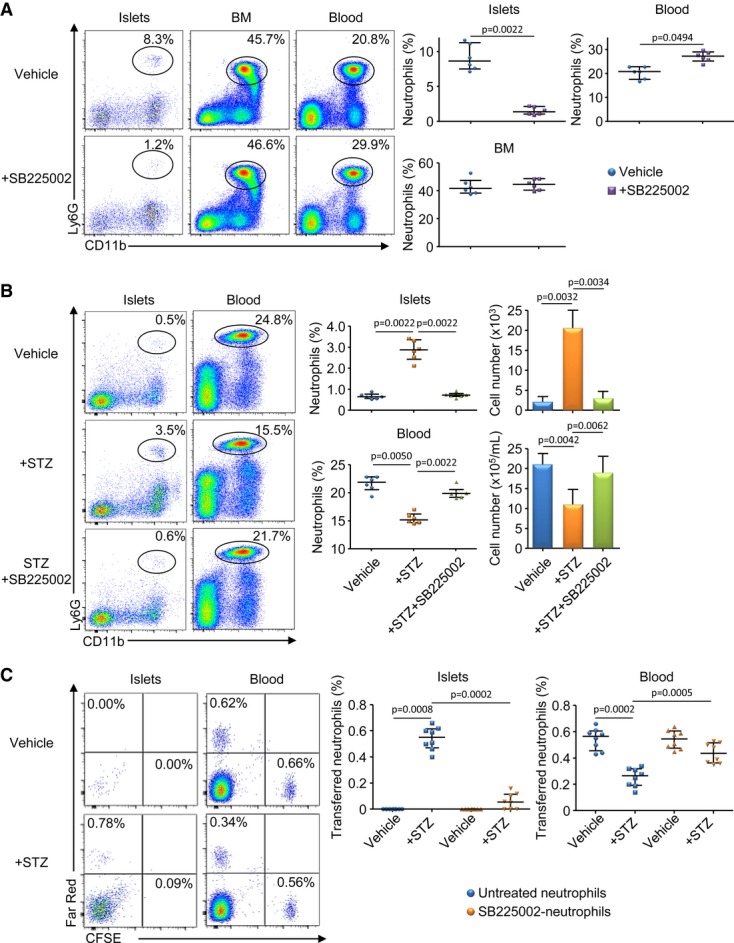
Neutrophils recruitment in the pancreas is dependent on CXCR2 A   Female NOD mice were treated or not between 1 and 3 weeks of age with the CXCR2 antagonist SB225002 (4 injections). Three days after the last treatment, cells were recovered from the pancreatic islets, BM, and blood and stained for CD45, CD11b, and Ly6G expressions. Frequency of neutrophils among CD45^+^ cells is represented. Data are representative (flow cytometry dot plot) or are median ± interquartile range (scatter plot) from three independent experiments with two independent pools of 2 mice for each group. B   Six-week-old female NOD mice were treated with SB225002 and, 12 h later, injected with streptozotocin (STZ). Twelve hours later, cells were harvested from the pancreatic islets and blood, and neutrophil frequency and absolute number among CD45^+^ population were measured. Data are representative (flow cytometry dot plot) or are median ± interquartile range (scatter and histogram plots) from three independent experiments with two independent mice for each group. C   Neutrophils were sorted from the BM of 6-week-old female NOD mice, and cells were treated *ex vivo* or not with SB225002. Then, treated and untreated neutrophils were stained with Cell trace® CSFE or FarRed, respectively, and cotransferred in 6-week-old NOD mice. Twelve hours after transfer, mice were injected with STZ, and 12 h after, cells were harvested from the pancreatic islets and blood and stained for CD45, CD11b, and Ly6G expressions. Data represented the frequency of transferred neutrophils among the neutrophil population. Data are representative (flow cytometry dot plot) or are median ± interquartile range (scatter plot) from eight independent experiments with four pooled mice for each group.

To investigate the role of CXCR2 in the recruitment of neutrophils in the pancreatic islets, we took advantage of the SB225002 molecule, a potent CXCR2 antagonist (White *et al*, 1998). After treatment in neonatal NOD mice, we observed that while CXCR2 blockade did not affect the frequency and absolute number of neutrophils in the BM, SB225002-treatment strongly prevented their recruitment from the blood to the pancreatic islets of 3-week-old NOD mice (Fig 2A and Supplementary Fig S1B). To confirm this result, we used the β-cell-specific toxic drug streptozotocin (STZ) that triggers a rapid recruitment of neutrophils to the pancreatic islets of 6-wk-old NOD mice (Diana *et al*, 2013). As expected, STZ injection induced an increased frequency and number of pancreatic neutrophils associated with a decreased frequency and absolute number of circulating neutrophils (Fig 2B). Importantly, this neutrophil trafficking was abrogated by CXCR2 blockade without affecting the recruitment of the other immune cells in the islets of STZ-treated mice (Supplementary Fig S3). We also observed *in vitro* that the addition of STZ to islet culture increased the production of CXCR2 ligands by the pancreatic islets, suggesting that β-cell stress induces chemokine release by the β-cells (Supplementary Fig S4). To exclude non-specific effect of the systemic injection of SB225002, we performed adoptive transfer experiments with neutrophils treated *ex vivo* with SB225002. CFSE-labeled neutrophils were treated *ex vivo* with SB225002, while FarRed-labeled neutrophils remained untreated before cotransfer in 6-week-old NOD mice. Then, NOD mice were treated with STZ, and we observed a decreased frequency and absolute number of FarRed^+^-untreated neutrophils among the blood neutrophil population and an increased frequency and absolute number of these cells among the newly recruited pancreatic neutrophil population (Fig 2C and Supplementary Fig S1C). On the contrary, CFSE^+^ SB225002-treated neutrophils failed to migrate from the blood to the pancreatic islets. We repeated these experiments using the reverse labeling to control for differential effects of dyes and obtained similar results (Supplementary Fig S5). Finally, a role for CCR1 in neutrophil recruitment to the pancreatic islets appeared unlikely as we failed to detect its ligands (CCL3 and CCL5) at 2 or 3 weeks of age in NOD mice (Supplementary Fig S6). Altogether, these data support a critical role for CXCR2 on the recruitment of neutrophils from the blood to the pancreatic islets in neonatal NOD mice.

### Neutrophil recruitment in the pancreatic islets is mediated by CXCR2 ligands

**Figure 3:**
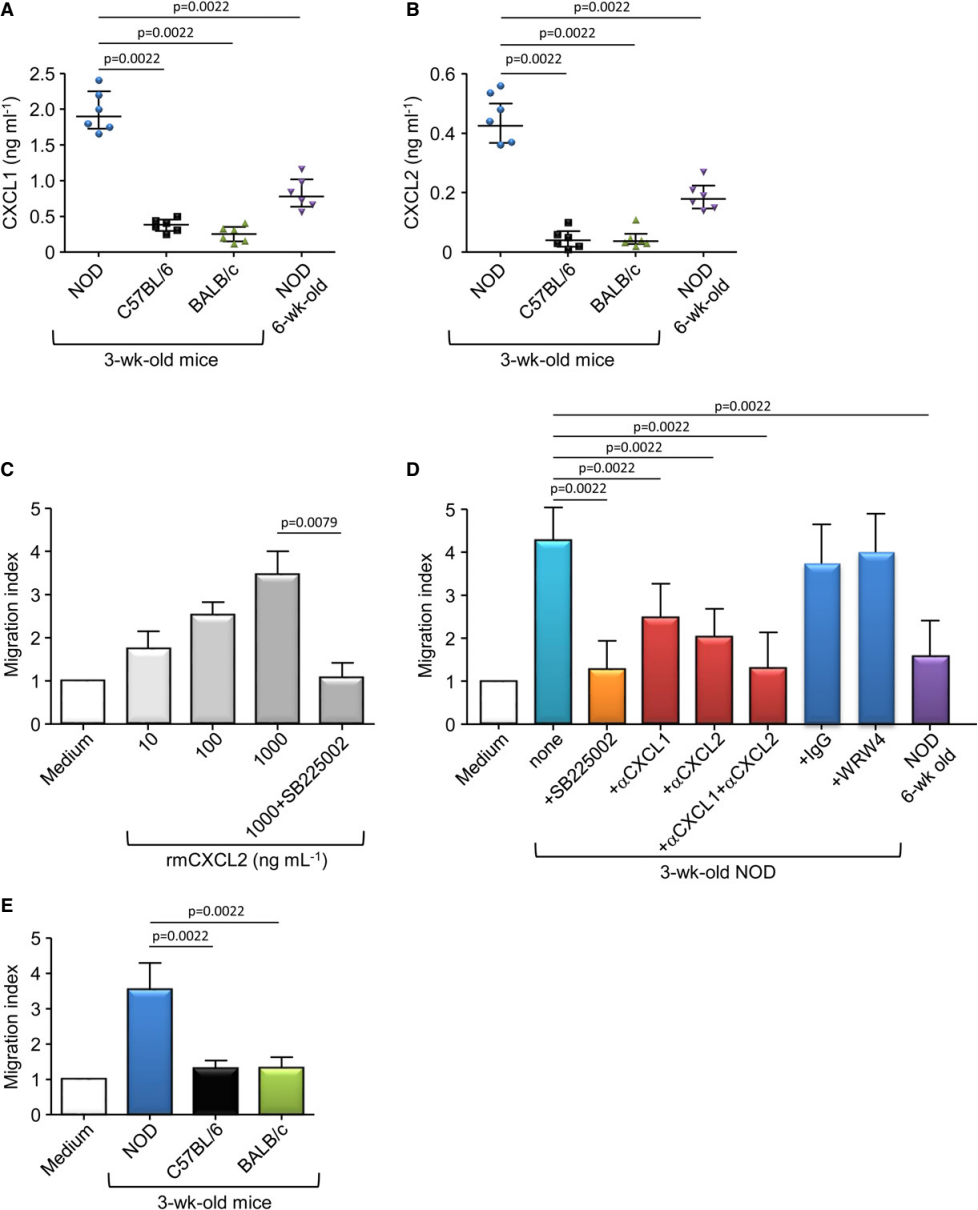
NOD pancreatic islets recruit neutrophils *in vitro* via CXCL1 and CXCL2 A, B   Pancreatic islets were isolated from female NOD, C57BL/6, and BALB/c mice at 3 or 6 weeks of age and cultured for 5 days. Then, CXCL1 and CXCL2 productions were measured by ELISA in the culture supernatants. Data are median ± interquartile range from three independent experiments with two independent mice for each group. C–E   Neutrophils were sorted from the blood of 6-wk-old female mice and used for *in vitro* migration assays toward various doses of rmCXCL2 (C), NOD-islet-conditioned media (D) or NOD-, C57BL/6-, or BALB/c-islet-conditioned media (E). In some conditions, neutrophils were pretreated with SB225002 or WRW4 before the migration assay (C, D), and in other conditions, neutralizing anti-CXCL1 or anti-CXCL2 pAbs were added in the islet-conditioned media (D). Data are migration index calculated assigning a value of 1 to the number of migrating cells toward medium alone. Data are median ± interquartile range from two (C), six (D), and five (E) independent experiments.

We next investigated whether CXCL1 and CXCL2 can be responsible for the neutrophil recruitment in the pancreatic islets as these chemokines are two high-affinity ligands of CXCR2 in mice (Eash *et al*, 2010; Schall & Proudfoot, 2011). Handpicked islets from 3-week-old NOD, C57BL/6 or BALB/c mice and from 6-week-old NOD mice were cultured, and the concentration in CXCR2 ligands in the islet-conditioned media was measured. We observed significantly higher amount of CXCL1 and CXCL2 released by the pancreatic islets from 3-week-old NOD mice compared with pancreatic islets from the two non-autoimmune mice or from 6-week-old NOD mice (Fig 3A and B). We next performed *in vitro* migration assays using blood neutrophils and islet-conditioned media as chemoattractant. As expected, blood neutrophils were efficiently attracted by rmCXCL2 and this migration was significantly abrogated by pretreatment of the neutrophils with CXCR2 antagonist (Fig 3C and Supplementary Table S1). Using islet-conditioned media, we observed that the migration of neutrophils was significantly higher using pancreatic islets from 3-week-old NOD mice compared with islets from 6-week-old NOD mice (Fig 3D and Supplementary Table S1). This migration was abrogated using SB225002-pretreated neutrophils or by the addition of antibodies against CXCL1 or CXCL2 in the islet-conditioned media; while pretreatment of neutrophils with the FPRL1 antagonist (WRW4) did not affect their recruitment (Fig 3D). Moreover, the neutrophil migration toward islet-conditioned media was NOD-specific as islet-conditioned media from the two non-autoimmune strains faintly attracted neutrophils (Fig 3E and Supplementary Table S1). Altogether, these data support that CXCL1 and CXCL2 were specifically produced in the pancreatic islets of 3-week-old NOD mice attracting neutrophils *in vitro*.

### CXCL1 and CXCL2 are produced *in vivo* by both β-cells and macrophages

**Figure 4:**
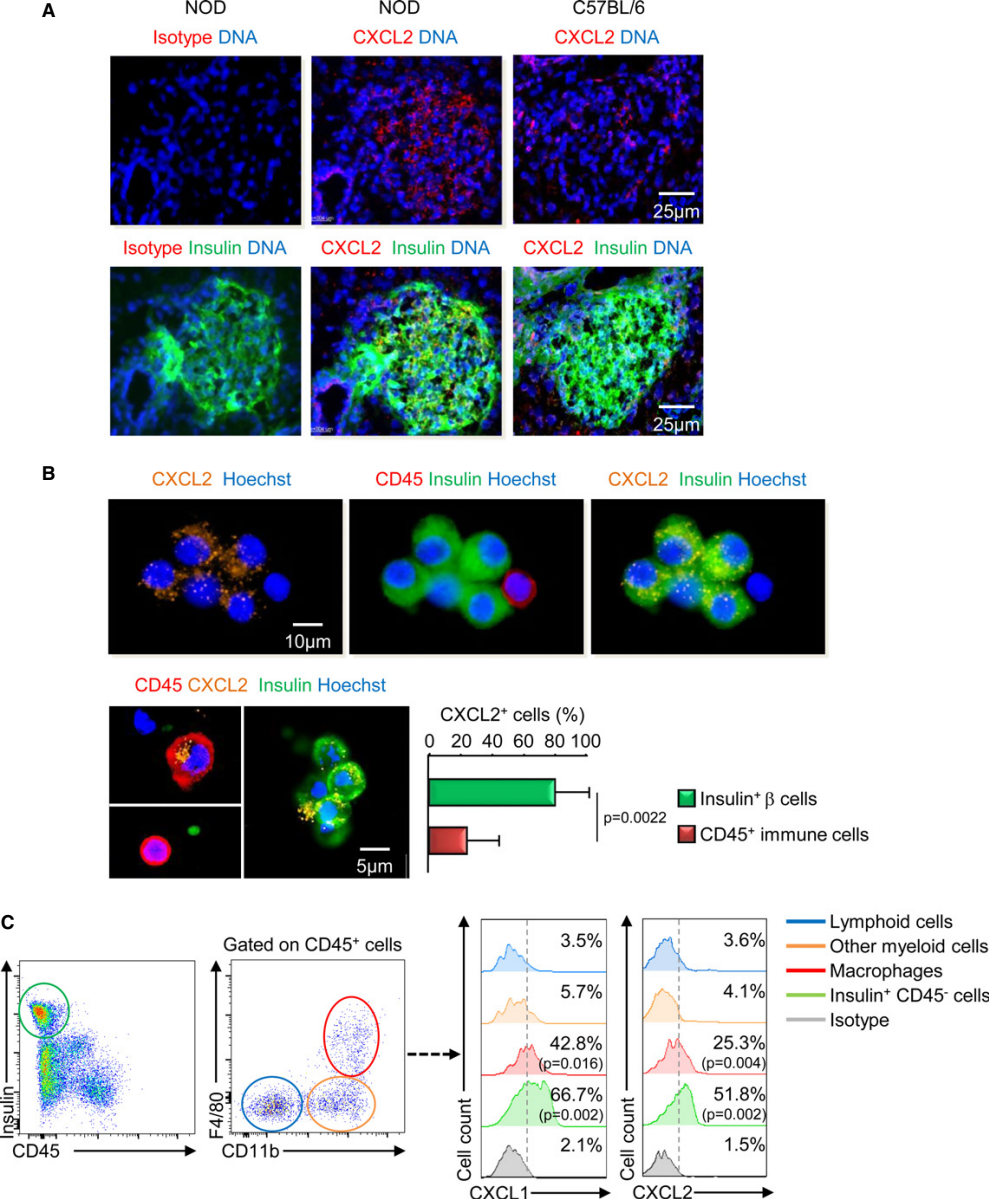
β-cells and macrophages produce CXCL1 and CXCL2 in the pancreatic islet of neonatal NOD mice A   Immunohistological analysis of pancreas from female NOD and C57BL/6 mice at 3 weeks of age. Pancreatic sections were prepared as described in Methods. Data are representative of three independent experiments with 2 pancreases per experiment with 10 sections for each pancreas. B   Pancreatic islet cell suspension were isolated and fixed for immunocytological analysis. Representative data from four independent mice are shown (picture), and the frequency of CXCL2^+^ cells among CD45^+^ or insulin^+^ cells is shown (histogram plot) (1,000 cells pooled from four pancreases were numbered). Data are representative (picture) or are median ± interquartile range (histogram plot) from four independent mice. C   Pancreatic islet cells were isolated from 3-week-old female NOD mice, and cells were incubated 5h in the presence of Brefeldin A before staining for CD45, CD11b, F4/80 surface expressions and for insulin and CXCL1 or CXCL2 intracellular expressions. Data are the frequency of CXCL1^+^ or CXCL2^+^ cells and are median ± interquartile range from three independent experiments with 2 pools of 2 mice per group for each experiment.

To investigate the cell source of CXCR2 ligands *in vivo*, we performed histological analysis of pancreas from neonatal NOD and C57BL/6 mice. We focused our analyses on CXCL2 because in our hands and as indicated by the manufacturer, the polyclonal antibody against mouse CXCL1 was not suitable for immunohistology. CXCL2 was present within the pancreatic islets and mainly colocalized with insulin, supporting that CXCL2 was produced by the β-cells (Fig 4A). Importantly, the expression was higher in the NOD mice compared with the C57BL/6 mice. As infiltrating immune cells could also produce CXCL2, we analyzed, by immunocytology, single-cell suspension from pancreatic islets of neonatal NOD mice. CXCL2 was produced by most of the insulin^+^ β-cells but also by some CD45^+^ immune cells (Fig 4B). We next performed flow cytometry analysis (Fig 4C) and observed that the CD45^−^ insulin^+^ β-cells highly expressed CXCL1 and CXCL2, confirming our immunohistological data, and that among the CD45^+^ immune cells, only macrophages (F4/80^+^ CD11b^+^) expressed CXCL1 and CXCL2 and no other myeloid cells (F4/80^−^ CD11b^+^) or lymphoid cells (CD11b^−^). Together, these data support that in the pancreatic islets of neonatal NOD mice, CXCL1 and CXCL2 were produced by both β-cells and macrophages.

### IL-1β is required for the production of CXCR2 ligands in the pancreatic islets

**Figure 5:**
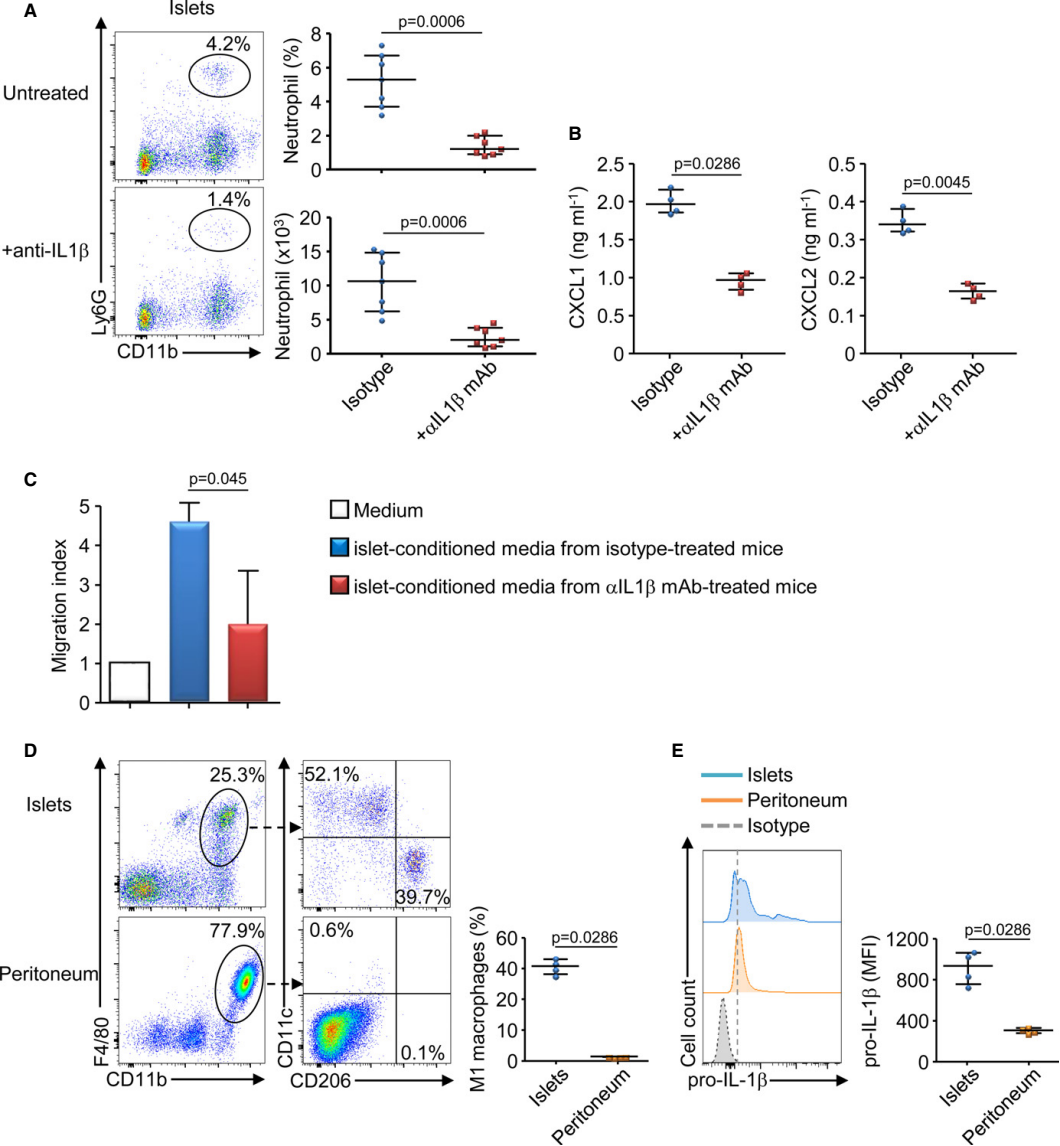
IL-1β is required for CXCR2 ligand production and neutrophil recruitment in the pancreatic islets A, B   10-day-old female NOD mice were treated with anti-IL1β mAb or isotype control, and 11 days after, pancreatic islets were analyzed for the presence of neutrophils by flow cytometry (A) or for CXCR2 ligands production by ELISA after culture (B). Data are representative (flow cytometry dot plot) or are median ± interquartile range (scatter plot) from seven (A) or four (B) independent experiments with three pooled mice per group. C   Neutrophils were sorted from the blood of 6-wk-old female mice and used for *in vitro* migration assays toward islet-conditioned media from untreated or anti-IL1β-treated 3-wk-old NOD mice. Data are median ± interquartile range from four independent experiments. D, E   Pancreatic islet cells or peritoneal cells were isolated from female NOD mice at 3 weeks of age, and cells were stained for CD45, CD11b, F4/80, CD11c, CD206 surface expressions and for pro-IL-1β intracellular expression. The frequency of M1 macrophages (CD11b^+^, F4/80^+^, CD11c^+^ CD206^−^) and the MFI of pro-IL-1β expression among macrophage population is represented. Data are representative (flow cytometry dot plot) or are median ± interquartile range (scatter plot) from four independent experiments with 3 pooled mice per group.

Then, we investigated the molecular pathway leading to the production of CXCR2 ligands by the β-cells. We focused on IL-1β as this proinflammatory cytokine stimulated the secretion of various chemokines by murine or human β-cells (Chen *et al*, 2001; Piemonti *et al*, 2002; Citro *et al*, 2012). We treated neonatal NOD mice with a neutralizing anti-IL-1β mAb, and we observed a significant decrease in the recruitment of neutrophils (Fig 5A) and in the production of CXCR2 ligands in the pancreatic islets (Fig 5B). Accordingly, *in vitro* neutrophil migration assays revealed that the chemotactic activity of islet-conditioned media from anti-IL-1β mAb-treated NOD mice was reduced compared with islet-conditioned media from isotype-treated NOD mice (Fig 5C). It is noteworthy that IL-1β blockade did not affect pancreatic infiltration by other myeloid cells at 3 weeks of age (Supplementary Fig S7). We investigated the cell source of IL-1β in the pancreatic islets of neonatal NOD mice, and among the CD45^+^ immune infiltrating cells, we noticed a high frequency of inflammatory M1 macrophages (F4/80^+^ CD11b^+^ CD11c^+^ CD206^−^) which were not detected in the peritoneum (Fig 5D). As M1 macrophages were known as proinflammatory cells, we analyzed their production of pro-IL-1β and we observed that pancreatic macrophages expressed significantly higher amount of pro-IL-1β compared with peritoneal macrophages (Fig 5E). We have chosen to analyze pro-IL1β expression by FACS analysis as it was technically difficult to isolate enough pancreatic macrophages to measure the secretion of the mature form of IL-1β by ELISA. These data support that in the pancreatic islets of neonatal NOD mice, inflammatory macrophages produced pro-IL-1β and that this cytokine was required for the production of CXCR2 ligands and the recruitment of neutrophils.

### IL-1β-secreting macrophages stimulate the production of CXCR2 ligands by the β-cells

**Figure 6:**
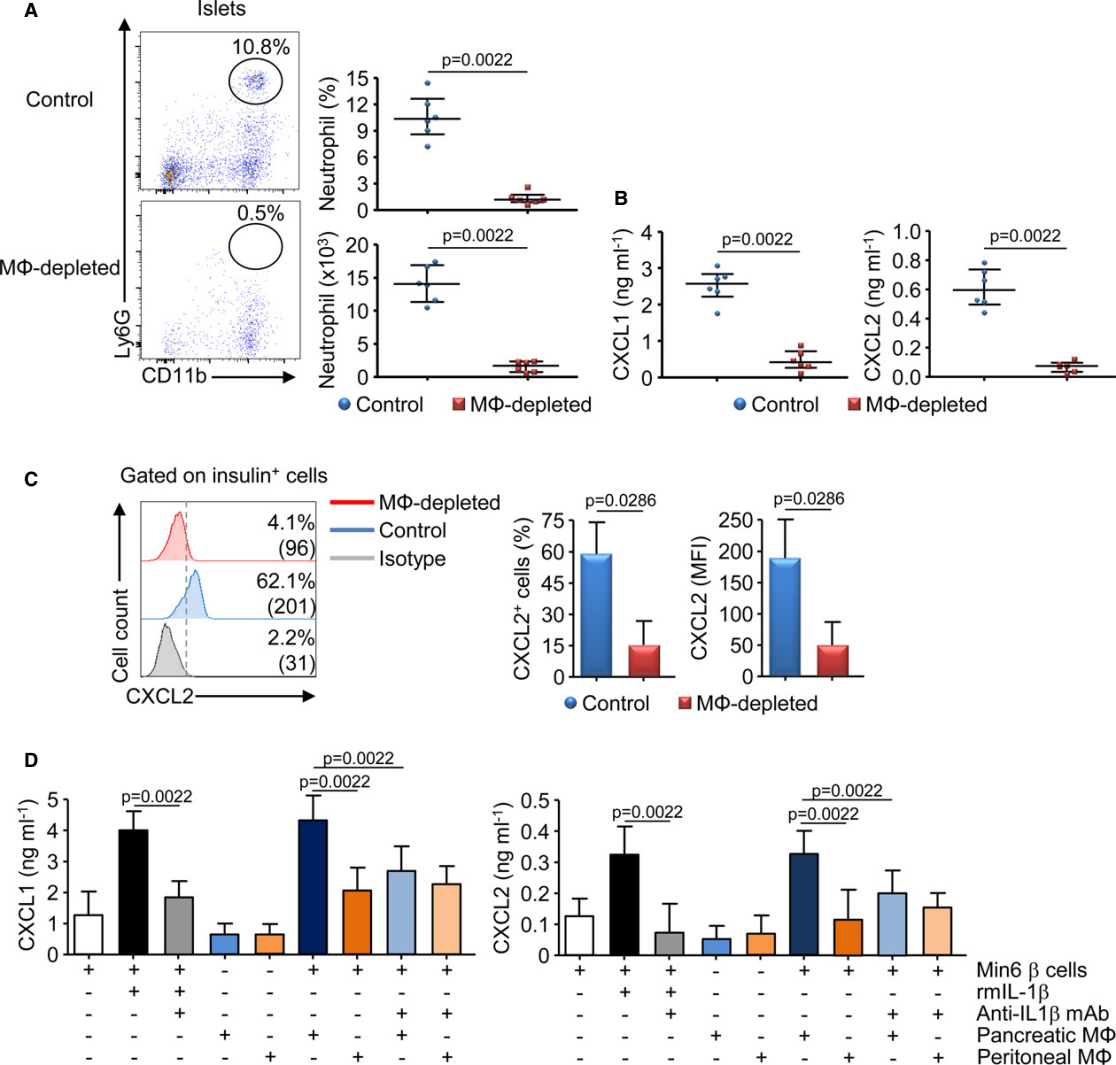
Pancreatic macrophages stimulate CXCR2 ligand production by the β-cells A–C   Macrophages were depleted by injection of clodronate liposomes or PBS liposomes (control) in female NOD mice between 1 and 3 weeks of age. Pancreatic islets were analyzed for the presence of neutrophils by flow cytometry (A), the production of CXCR2 ligands after culture by ELISA (B), or the expression of CXCL2 by the β-cells by flow cytometry (C). Data are representative (flow cytometry plot) or are median ± interquartile range (histogram or scatter plot) from three independent experiments each performed with two independent pools of three mice per group. D   Pancreatic IL-1β-producing macrophages stimulate CXCL1/2 production by β-cells *ex vivo*. Min6 β-cells and sorted pancreatic or peritoneal macrophages from 3-week-old female NOD mice were cocultured for 48 h in a Transwell system. CXCL1 and CXCL2 productions in the culture supernatant were measured by ELISA. Data are median ± interquartile range from four independent experiments.

To directly address the role of macrophages in the recruitment of neutrophils in the pancreatic islets, we performed depleting experiments using clodronate-liposome injection in neonatal NOD mice. We observed that macrophage depletion resulted in a significant reduction in immune cell infiltration, particularly of neutrophils, associated with a decreased production of CXCR2 ligands in the pancreatic islets (Fig 6A and B). Then, by flow cytometry analysis, we observed that macrophage depletion strongly diminished the expression of CXCL2 (frequency and MFI) by the insulin^+^ β-cells (Fig 6C). To further show that pancreatic IL-1β-secreting macrophages stimulated the β-cells to produce CXCR2 ligands, we performed *in vitro* experiments using the Min6 β-cell line cultured in a Transwell system with macrophages isolated from the pancreatic islets or the peritoneum of 3-week-old NOD mice (Fig 6D). First, we validated that Min6 β-cells expressed CXCL2 and that such expression was increased by the presence of rmIL-1β (Supplementary Fig S8). We observed that macrophages and Min6 β-cells cultured separately were able to produce low level of CXCR2 ligands. However, similarly to rmIL-1β, the addition of pancreatic macrophages but not of peritoneal macrophages increased the production of CXCR2 ligands in the β-cell culture (Fig 6D). As we did not observed, by flow cytometry analysis, an increased expression of CXCL2 by pancreatic macrophages after culture with the β-cells (Supplementary Fig S9), we concluded that the β-cells were responsible for the increased production of chemokines observed in the coculture with pancreatic macrophages. Finally, these productions of CXCR2 ligands were abrogated by the addition of anti-IL1β mAb in the coculture. Altogether, our *in vivo* and *in vitro* data strongly support that IL-1β released by pancreatic macrophages stimulated the production of CXCR2 ligands by the β-cells.

### Blockade of neutrophil recruitment in the pancreatic islets dampened T1D development

**Figure 7:**
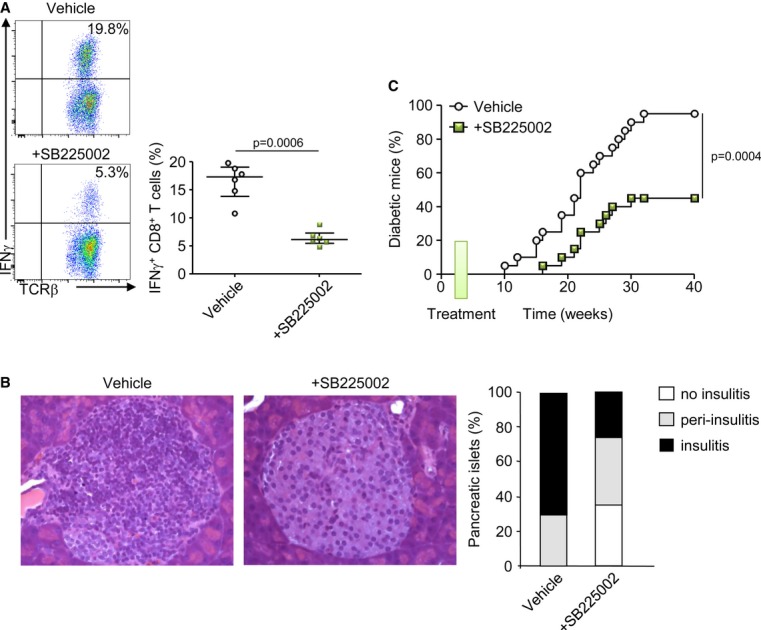
Early blockade of neutrophil recruitment dampens the diabetogenic process in the NOD mice A   Analysis of CD8^+^ T cells specific for IGRP_206–214_ in 8-week-old female NOD mice after SB225002 treatment or vehicle between 1 and 3 weeks of age (4 injections). Islets were harvested and cultured for 8 days, and cells were stimulated for 5 h with IGRP_206–214_ peptide. Values are the frequency of IFN-γ^+^ cells among CD8^+^ T cells. Data are representative (flow cytometry dot plot) or are median ± interquartile range (scatter plot) from three independent experiments with two independent mice for each. B   Histological analysis of pancreatic islet sections stained with hematoxylin and eosin from 12-week-old female NOD mice treated or not with SB225002 between 1 and 3 weeks of age (*n* = 3 mice per group). C   Female NOD mice were treated with SB225002 or vehicle between 1 and 3 weeks of age (4 injections), and incidence of diabetes was followed. *N* = 16 mice/group.

We have recently demonstrated that neutrophils participate to the initiation of the diabetogenic process (Diana *et al*, 2013). We speculated that the blockade of neutrophil recruitment into the pancreatic islets would affect T1D development in NOD mice. We evaluated the well-defined autoreactive CD8 T-cell response against the β-cell antigen islet-specific glucose-6-phosphatase catalytic subunit-related protein (IGRP)_206-214_ in 8-wk-old NOD mice that were previously treated with SB225002 between 1 and 3 weeks of age. We observed that early CXCR2 blockade led to a dramatic reduction in IFN-γ^+^ IGRP_206-214_-reactive CD8^+^ T cells within the islets (Fig 7A) and accordingly, dampened the infiltration of the islets and the development of autoimmune diabetes up to 40 weeks of age in the NOD mice (Fig 7B and C). It is noteworthy that CXCR2 blockage at later age (between 8 and 10 weeks of age) did not affect subsequent diabetes development (Supplementary Fig S10). These data support that the blockage of neutrophil recruitment in the islets of neonatal NOD mice reduced the later development of T1D development.

## Discussion

We have recently reported that neutrophils play a major role in the initiation of autoimmune diabetes in the NOD mice (Diana *et al*, 2013). Here, we demonstrate that the recruitment of neutrophils from the blood to the pancreatic islets of NOD mice is dependent on CXCR2. We further show that the two CXCR2 ligands CXCL1 and CXCL2 are produced by both pancreatic macrophages and the β-cells. Furthermore, our data demonstrate that IL-1β secreted by inflammatory macrophages stimulates the production of CXCR2 ligands by the β-cells. Importantly, the blockade of neutrophils recruitment in the neonatal NOD mice using a CXCR2 antagonist dampens the diabetogenic T-cell response, the insulitis, and the incidence of diabetes. These data reveal a novel crosstalk between inflammatory macrophages, β-cells, and neutrophils and support that in an autoimmune-prone genetic background, the β-cells actively participate to their own demise by recruiting diabetogenic neutrophils. The presence of neutrophils in the target tissue appears as a common trait in the pathogenesis of various autoimmune or autoinflammatory diseases (Mocsai, 2013). Importantly, our recent findings in the NOD mice appear to be relevant to the human disease as a recent publication shows that a peripheral neutropenia precedes T1D onset in patients and that neutrophils are observed in the islets of multi-organ donors with recent-onset T1D (Valle *et al*, 2013).

The trafficking of infiltrating immune cells during the development of autoimmune diabetes has been investigated notably using chemokine receptor knockout mice or transgenic animal overexpressing specific chemokine under the control of the insulin promoter. Several chemokines has been detected in the pancreatic islets during the progression of the disease, allowing the recruitment of myeloid cells through CCR2 and CCR5 and of lymphoid cells through CXCR3 and CCR5 (Eizirik *et al*, 2009). However, these chemokine receptors are responsible for the recruitment of both diabetogenic and protective cells as revealed by the reduced incidence of diabetes observed in the CCR2^−/−^ NOD mice and the accelerated incidence in the CXCR3^−/−^ or the CCR5^−/−^ NOD mice (Solomon *et al*, 2010; Yamada *et al*, 2012). Neutrophil trafficking from the BM into the circulation and from the blood into the inflamed peripheral tissue is a tightly controlled phenomenon avoiding collateral tissue damage due to the release by activated neutrophils of various toxic molecules (Sadik *et al*, 2011; Amulic *et al*, 2012). CXCR2 ligands are critical for the recruitment of neutrophils in the tissue, and depending on the pathological context, other chemokine receptors also play a role (Sadik *et al*, 2011). During autoantibody-induced arthritis, the recruitment of neutrophils in the inflamed joint requires a cascade of cytokines and chemokines (LTB4, IL-1β, CCR1, and CXCR2 ligands) produced by endothelial cells, synovial cells, and neutrophils themselves (Chen *et al*, 2006; Kim *et al*, 2006; Chou *et al*, 2010; Sadik *et al*, 2011). In our study, we demonstrate that the infiltration of the pancreatic islets by the neutrophils requires CXCR2 and that its ligands CXCL1 and CXCL2 are produced in part by the β-cells in the target tissue. One should consider that other chemokines targeting CXCR2 may be produced by the β-cells and play additional role in the recruitment of neutrophils in the pancreatic islets. A pioneer study has demonstrated that mouse β-cells are directly responsible for the recruitment of diabetogenic CXCR3^+^ T cells in a virally induced model of T1D (Frigerio *et al*, 2002). We also observed that pancreatic macrophages produce CXCR2 ligands as previously described (Sadik *et al*, 2011, 2012). Importantly, we show that macrophages also produced CXCR2 ligands in the pancreatic islets. To define the precise contribution of each cell type in the production of CXCR2 ligands and in the subsequent recruitment of neutrophils in the pancreatic islets, a selective and conditional knocking out of these chemokines specifically in the β-cells may be a powerful tool.

We excluded a putative role for cathelicidin in neutrophil recruitment (De *et al*, 2000), as blocking of its receptor (FPRL1) on neutrophils using WRW4 antagonist did not affect their *in vitro* recruitment. Importantly, a recent study has demonstrated a critical role for CXCR2 in the rejection of islet graft both in alloxan-induced diabetic BALB/c mice and in T1D patients (Citro *et al*, 2012). In mice, the authors demonstrated that grafted islets produce CXCR1/2 ligands stimulating the recruitment of harmful polymorphonuclear leukocytes in the transplanted islets and that CXCR1/2 blockade delays islet rejection. In humans, they demonstrated that the CXCR1/2 blockade improves islet transplantation outcome in T1D patients. These data and ours strongly support that the targeting of neutrophil recruitment *via* CXCR2 blockade may be therapeutically pertinent to prevent pancreatic islet destruction during T1D development or after islet transplantation.

We demonstrate that β-cells produce CXCR2 ligands in response to IL-1β secreted by inflammatory macrophages. It has been previously shown in both rodents and humans that β-cells are high producers of chemokines in response to inflammatory cytokines (Cardozo *et al*, 2003; Ehses *et al*, 2009; Jacobsen *et al*, 2009; Sarkar *et al*, 2012). Indeed, β-cells are uniquely susceptible to IL-1β as they harbor a higher expression of IL-1R1 compared with other cells from various tissues (Boni-Schnetzler *et al*, 2009). In our study, treatment with anti-IL1β mAb in neonatal NOD mice dampens the production of CXCL1 and CXCL2 and the recruitment of neutrophils into the pancreatic islets. Regarding the role of neutrophils in the initiation of the disease, one could expect that such IL-1β blockade at early ages may reduce the development of diabetes in NOD mice. Remarkably, some authors have reported that treatment with soluble IL-1 receptor or neutralizing anti-IL-1β pAb prevents cyclophosphamide-induced diabetes in NOD mice (Nicoletti *et al*, 1994; Cailleau *et al*, 1997). A recent study also shows a synergistic reversal of diabetes in NOD mice with anti-CD3 antibody and IL-1 blockade (Ablamunits *et al*, 2012). However, other groups have shown that IL-1R deficiency only slows the progression of spontaneous T1D in NOD mice and that IL-1R antagonist treatment of diabetic NOD mice fails to reverse the disease (Thomas *et al*, 2004). In our study, IL-1β blockade did not affect pancreatic infiltration by other immune cells at 3 weeks of age supporting that IL-1β is not the only cytokine responsible for the pancreatic inflammation during T1D development. Paradoxically, we observed an only transient expression of CXCR2 ligands in the neonatal NOD mice even though proinflammatory cytokines remain present in the pancreatic islets of older NOD mice. One could hypothesize that the β-cells produce various chemokines at each step of T1D development according to the type of infiltrating cells in the pancreatic islets. Moreover, it has been shown that type I IFN dampens neutrophil recruitment and the production of CXCR2 ligands in a septic peritonitis model and after influenza virus infection (Shahangian *et al*, 2009; Kelly-Scumpia *et al*, 2010). As we have previously showed that pancreatic neutrophils stimulate the production of type I IFN by pDCs (Diana *et al*, 2013), we hypothesize that neutrophils could indirectly downregulate their own recruitment into the pancreatic islets.

We demonstrate that in neonatal NOD mice, pancreatic islets are infiltrated by inflammatory macrophages producing CXCR2 ligands and pro-IL-1β stimulating the production of CXCR2 ligands by the β-cells. It has been described that macrophages are the first cells to infiltrate the islets during diabetes development (Dahlen *et al*, 1998). Indeed, during the first postnatal weeks, waves of physiological β-cell death occur in rodents (Trudeau *et al*, 2000; Mathis *et al*, 2001), pigs (Bock *et al*, 2003) and humans (Kassem *et al*, 2000) leading to the recruitment of macrophages required to eliminate cell debris. In the NOD genetic background, these macrophages produce exaggerated amounts of inflammatory cytokines leading to a persistent inflammation of the pancreas (Stoffels *et al*, 2004). Remarkably, this genetic defect of macrophages has been also identified in human with monocytes isolated from the blood of T1D patients (Devaraj *et al*, 2006; Bradshaw *et al*, 2009).

Altogether, these data reveal a novel crosstalk in the pancreatic islets between macrophages, β-cells, and neutrophils during the first postnatal weeks in the NOD mice. This study provides a new example that in an autoimmune-prone genetic background, pancreatic β-cells actively participate to their own demise as recently discussed by Atkinson *et al* (Atkinson *et al*, 2011).

## Materials and methods

### Mice and treatments

Female BALB/c, C57BL/6, and NOD mice were bred and housed in specific pathogen-free conditions. For CXCR2 blocking, mice were intraperitoneally (i.p.) injected with SB225002 (0.5 mg/kg, Calbiochem) or vehicle (PBS-1% ethanol) between 1 and 3 weeks of age (4 injections). For induction of β-cell death, 6-week-old mice were injected once i.p. with streptozotocin (80 mg/kg, Sigma) or vehicle (citrate buffer) as previously described (Turley *et al*, 2003). For IL-1β blocking, mice were injected i.p. with anti-IL-1β mAb (0.2 mg/mouse, 01BSUR, Novartis Pharma AG) or isotype control (rat IgG1) between 1 and 3 weeks of age (4 injections). To deplete macrophages, mice were injected i.p. with 200 μL of clodronate- or PBS-loaded liposomes (control) between 1 and 3 weeks of age (4 injections) as previously described (Van Rooijen & Sanders, 1994) (from ClodronateLiposomes.com). All animal experimental protocols were approved by the ethic committee for animal experimentation (CEEA34.JD.046.12).

### Preparation of pancreatic islets

Pancreata were perfused with 3 mL of a solution of collagenase P (1.5 mg/mL, Roche) and then dissected free from surrounding tissues. Pancreata were digested at 37°C for 10 min. Digestion was stopped by adding HBSS-5% FCS followed by extensive washes. For flow cytometry analysis, islets were purified on a discontinuous Ficoll gradient, and for islet cultures, islets were purified by handpicking to avoid contamination by exocrine tissue.

### Flow cytometry

Single-cell suspensions were prepared from various tissues and were stained for 30 min at 4°C after FcγRII/III blocking in PBS containing 2% FCS, 0.5% EDTA, and 0.1% sodium azide. Surface staining was performed with the following antibodies: anti-CD45 (eBioscience, 30-F11), -CD11b (eBioscience, M1/70), -Ly6G (BD, 1A8), -CXCR4 (eBioscience, 2B11), -CXCR2 (Biolegend, TG11), -F4/80 (eBioscience, BM8), -CD11c (eBioscience, N418), and -CD206 (Biolegend, C068C2) mAbs. For intracellular CXCL1, CXCL2, and pro-IL-1β staining, cell suspension was incubated 5 h at 37°C with Brefeldin A (Sigma). After fixation and permeabilization (BD Fix&Perm®), cells were stained with anti-mouse CXCL1 or CXCL2 pAbs (R&D) and F(ab')2 donkey anti-rabbit-PE pAb (eBioscience) or with anti-mouse IL-1β Pro-form mAb (eBioscience, IL-1F2). For analysis of insulin^+^ cells, pancreatic islet cells were surface-stained with anti-CD45 mAbs, then after fixation and permeabilization (BD Fix&Perm®), cells were stained with anti-insulin pAb (Abcam) following by secondary antibody staining (anti-guinea pig-AlexaFluor488 pAb, Invitrogen). For measurement of diabetogenic CD8^+^ T-cell response, handpicked islets were cultured for 7 days in the presence of rmIL-2 (R&D, 50 U/mL). Then isolated cells were stimulated for 4 h at 37°C with bone marrow-derived DCs loaded with IGRP_206–214_ peptide in the presence of Brefeldin A before surface staining with TCRβ (eBioscience, H57-597) and CD8 (BD, 53–6.7) mAbs and intracellular staining with anti-IFN-γ (eBioscience, XMG1.2) mAb. In all experiments, dead cells were excluded using Fixable Viability Dye (eBioscience). Stained cells were analyzed and/or sorted on a BD FACS Aria II flow cytometer.

### Neutrophil transfer

Neutrophils were isolated from the bone marrow (BM) of 6-week-old NOD mice using cell sorting according to their positive expression of CD45, CD11b, and Ly6G. The purity of sorted neutrophil population was routinely between 98 and 99%. Then, neutrophils were treated or not with SB225002 (100 nM) or diluent (PBS-1% ethanol) for 10 min at 37°C. After extensive washes, treated or untreated cells were stained with CellTrace® CFSE or FarRed (Invitrogen), respectively. A mix (1:1) of treated and untreated neutrophils (total 20 x 10^6^ cells) was injected (i.v.) in 6-week-old NOD mice. Twelve hours after transfer, mice were treated with streptozotocin or vehicle, and after 12 h, transferred cells were analyzed in the blood and the islets.

### CXCL1 and CXCL2 measurements

CXCL1 and CXCL2 production was evaluated in handpicked islet culture and in cell culture supernatants by ELISA according to the manufacturer protocols (Sigma). One hundred islets were cultured for 5 days in DMEM 10% FBS and 1% penicillin/streptomycin, and cell-free supernatants were recovered after centrifugation.

### *In vitro* migration assay

Neutrophils were isolated from the blood of 6-week-old NOD mice using cell sorting according to the positive expression of CD45, CD11b, and Ly6G. The purity of sorted neutrophil population was routinely between 98 and 99%. Chemotaxis was assayed in 24-well micro-chemotaxis chambers (Costar) using 5-μm PVP-free polycarbonate filter. Neutrophils (10^5^ cells/100 μL) were allowed to migrate toward various doses of rmCXCL2 (R&D), islet supernatant (100 islets equivalent), or medium alone (final volume: 600 μL). In some conditions, neutrophils were pre-treated with SB225002 (100 nM) or WRW4 (10 mM, Tocris) for 15 min at 37°C and neutralizing anti-CXCL1 or anti-CXCL2 mAbs (100 μg/mL, R&D) or rat IgG (100 μg/mL) as control was added to the islet supernatants 30 min prior to migration assay. After 4 h at 37°C, migrating cells were recovered in the lower compartment of the chamber and numbered by flow cytometry. Each sample was assayed in triplicate. Results are expressed as migration index: number of migrating neutrophils in a defined condition divided by the number of migrating neutrophils toward medium alone.

### *In vitro* coculture of β-cells and pancreatic macrophages

Culture was performed using tissue culture inserts (0.2 μm). Sorted pancreatic macrophages (2 x 10^4^ cells, CD45^+^ CD11b^+^ F4/80^+^) were added to the upper compartment, and 10^6^ Min6 β-cells were added in the lower compartment. In some conditions, Min6 β-cells were stimulated by the addition of rmIL-1β (10 ng/mL, Miltenyi) in the upper compartment. In some conditions, neutralizing anti-IL-1β mAb (Novartis Pharma AG) was added in the lower compartment (200 μg/mL). Cells were cultured for 2 days, the cell-free supernatant of the lower compartment was recovered, and CXCL1 and CXCL2 productions were measured by ELISA.

### Histology and cytology

Pancreases from NOD mice were embedded in tissue-freezing medium (Jung) and stored at −20°C. Tissues were cut into 5-μm section in cryostat (Leica). Frozen sections were fixed in cold acetone. The ability of islet-infiltrating cells to produce CXCL2 was assayed as follows. Cells were seeded on SuperFrost Plus microscope slide (Menzel-Glaser) at a concentration of 10^6^ cells/mL for 1 h at 37°C in DMEM, 10% FBS and 1% penicillin/streptomycin. Then, cells were fixed and then stained with anti-CXCL2 pAb (R&D) and anti-CD45-APC mAb and then anti-insulin pAb, each for 30 min. After washing, second-step reagents were applied: anti-rabbit-AlexaFluor555 and anti-guinea pig-AlexaFluor488 pAbs (Invitrogen), respectively. Nucleuses were stained with Hoechst. Image acquisition was performed on the Cochin Imaging Facility using a microscope Leica DMI6000.

### Diabetes diagnosis and histology

NOD females were tested every week for diabetes. Overt diabetes was defined as two positive urine glucose tests, confirmed by a glycemia > 200 mg dL^−1^. Glukotest kit was purchased from Roche. For histology analysis, paraffin-embedded sections were cut and stained with hematoxylin–eosin. Insulitis severity was scored with at least 40 islets per mouse analyzed from 12-week-old mice.

### Statistical analysis

Diabetes incidence was plotted according to the Kaplan–Meier method. Incidences between groups were compared with the log-rank test. For other experiments, reported values are median ± interquartile range as indicated. Comparison between medians was performed using the nonparametric Mann–Whitney *U*-test. *P* values < 0.05 were considered statistically significant. All data were analyzed using GraphPad Prism v5 software.

**The paper explained**

**Problem**

The incidence of T1D has dramatically increased worldwide in recent decades at a rate of 3–5% per year that is doubling approximately every 20 years. The incidence of T1D has also risen remarkably in very young children, and T1D can be seen more frequently in subjects who express low-risk alleles. Studies in animal models, particularly in the non-obese diabetic (NOD) mice, have identified several mechanisms involved in the different steps of T1D. These recent progresses in our understanding of T1D led to over a dozen novel therapies being tested in T1D patients. However, results from clinical trials have so far been disappointing, and there are still many unanswered questions that need to be addressed to identify new therapeutic targets to prevent or cure the disease. T1D development results from the interaction between pancreatic β-cells and the innate and the adaptive immune systems culminating with the destruction of the insulin-secreting β-cells by autoreactive T cells. The diabetogenic course starts during the first postnatal weeks by the infiltration of the pancreas by innate immune cells and especially neutrophils as recently shown by us. Here, we aim to determine the cellular and molecular mechanism leading to the recruitment of the diabetogenic neutrophils in the pancreas of neonatal NOD mice.

**Results**

Here, we show that neutrophils infiltrate the pancreas of neonatal NOD mice while they were not detected in the non-autoimmune-prone C57BL/6 and BALB/c mice. Neutrophil recruitment from the blood to the pancreas requires CXCR2 expression on the neutrophils and the production of CXCR2 ligands (CXCL1 and CXCL2) in the pancreas of neonatal NOD mice.

More particularly, in the pancreas, CXCR2 ligands are produced by both the insulin-producing β-cells and the infiltrating inflammatory macrophages. Pancreatic macrophages also produce the inflammatory cytokine IL-1β stimulating the production of CXCR2 ligands by the β-cells. Importantly, the blockade of CXCR2 by specific inhibitor in neonatal NOD mice reduces the diabetogenic T-cell response, the insulitis, and the incidence of diabetes.

**Impact**

To our knowledge, our study reveals for the first time the interaction between inflammatory macrophages and β-cells leading to the recruitment of diabetogenic neutrophils in the pancreas of neonatal NOD mice. This study supports that the use of blocking agents to inhibit the recruitment of neutrophils in the pancreas may be a promising therapeutic approach to prevent spontaneous autoimmune diabetes or to preserve pancreatic islet graft.

## Supporting information

for this article is available online: http://embomolmed.embopress.org
