# Supplementary material for: Macrophages and β-cells are responsible for CXCR2-mediated neutrophil infiltration of the pancreas during autoimmune diabetes
Source: EMBO Mol Med. 2014 Jun 26;6(8):1090–104. doi: 10.15252/emmm.201404144 (PMC4154135; doi:10.15252/emmm.201404144)
Supplement: Supplementary file 1 [file emmm0006-1090-sd1.pdf]

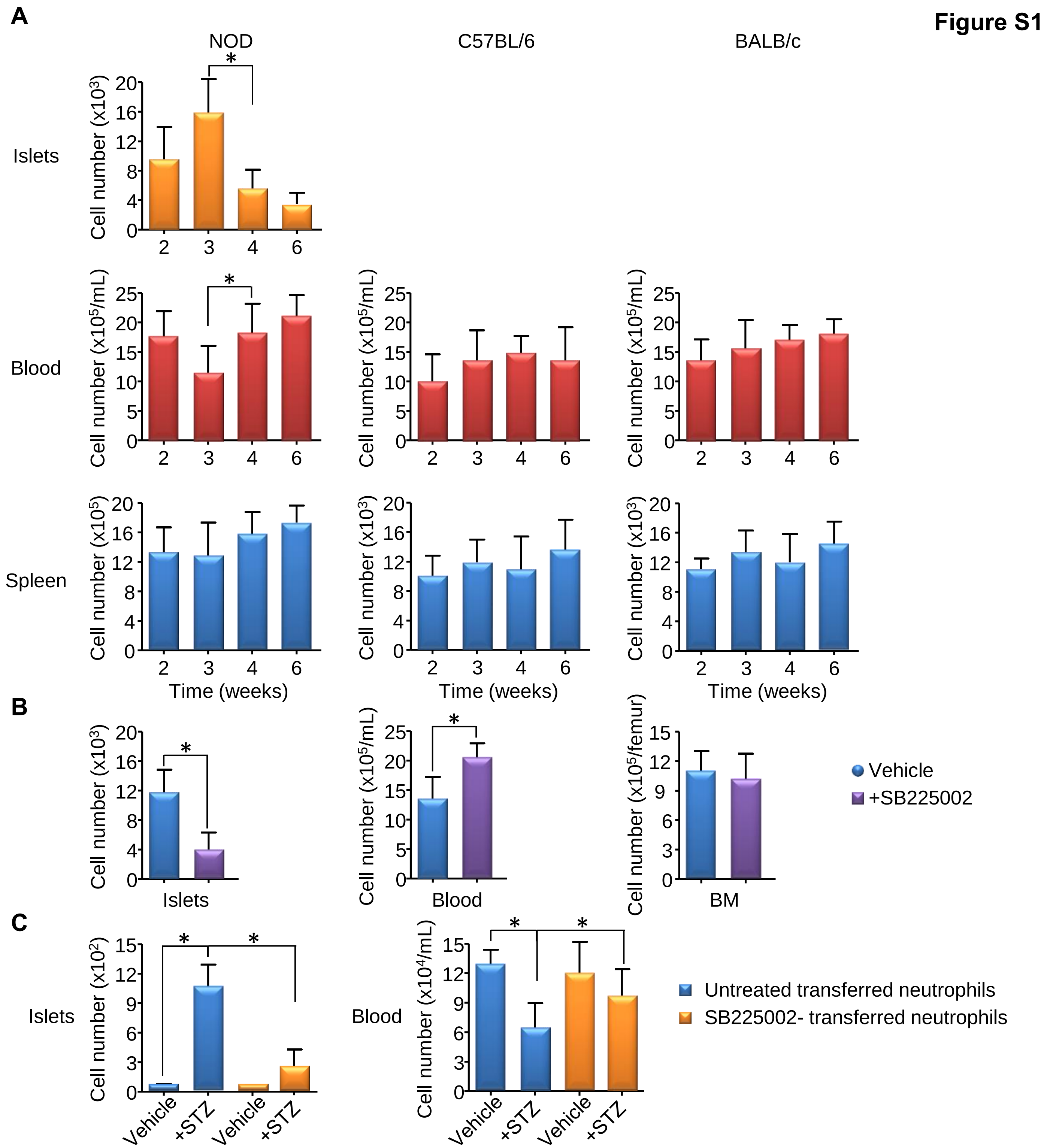

FigureS1. Absolute number of neutrophils from NOD, C57BL/6 and BALB/c mice. (a) Kinetic analysis of neutrophils in the pancreatic islets, blood and spleen of NOD, C57BL/6 and BALB/c mice. Cells were harvested on various weeks of age and stained for CD45, CD11b and Ly6G expressions. Data are median +/– interquartile range from four independent experiments with three pooled mice for each group. *: $P < 0.05$ for each group compared to 2-wk-old group. **(b)** Analysis of neutrophils in the pancreatic islets, blood and bone marrow of NOD after SB225002 treatment or after neutrophil transfer **(c)**. Cells were harvested and stained for CD45, CD11b and Ly6G expressions. . Data are median +/– interquartile range from three independent experiments with two independent mice for each group. *: $P < 0.05$.
