# Supplementary material for: Macrophages and β-cells are responsible for CXCR2-mediated neutrophil infiltration of the pancreas during autoimmune diabetes
Source: EMBO Mol Med. 2014 Jun 26;6(8):1090–104. doi: 10.15252/emmm.201404144 (PMC4154135; doi:10.15252/emmm.201404144)
Supplement: Supplementary file 2 [file emmm0006-1090-sd2.pdf]

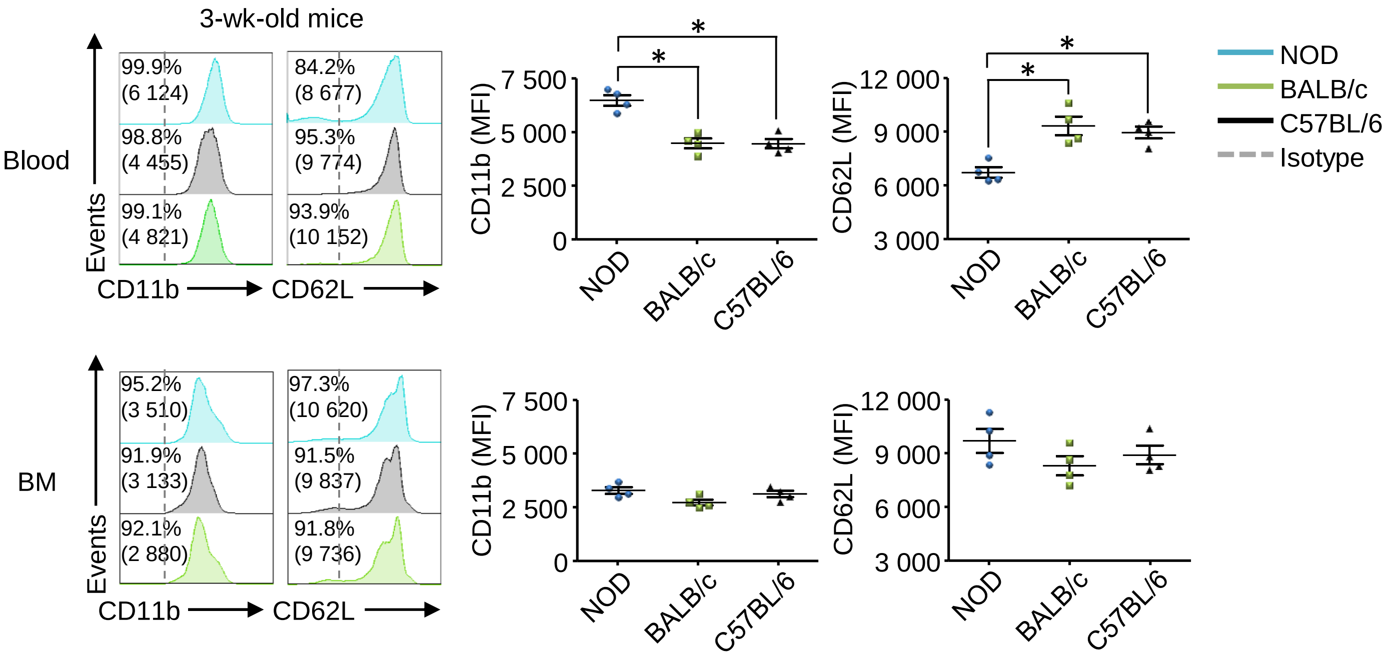

Figure S2. Phenotype of neutrophils from NOD, C57BL/6 and BALB/c mice at 3wks of age. Cells from the blood and bone marrow (BM) were recovered from NOD, C57BL/6 and BALB/c mice and stained for CD45, CD11b, Ly6G, and CD62L expressions. The percentage of positive cells and the mean fluorescence intensity (MFI) of CD11b and CD62L on neutrophils are represented. Dotted line represents the staining with control isotype. Data are representative (flow cytometry histogram) or are mean values +/– SEM (scatter plot) from 2 independent experiments with two independent mice for each group. *: P < 0.05 comparing group as indicated.
