# Supplementary material for: Macrophages and β-cells are responsible for CXCR2-mediated neutrophil infiltration of the pancreas during autoimmune diabetes
Source: EMBO Mol Med. 2014 Jun 26;6(8):1090–104. doi: 10.15252/emmm.201404144 (PMC4154135; doi:10.15252/emmm.201404144)
Supplement: Supplementary file 3 [file emmm0006-1090-sd3.pdf]

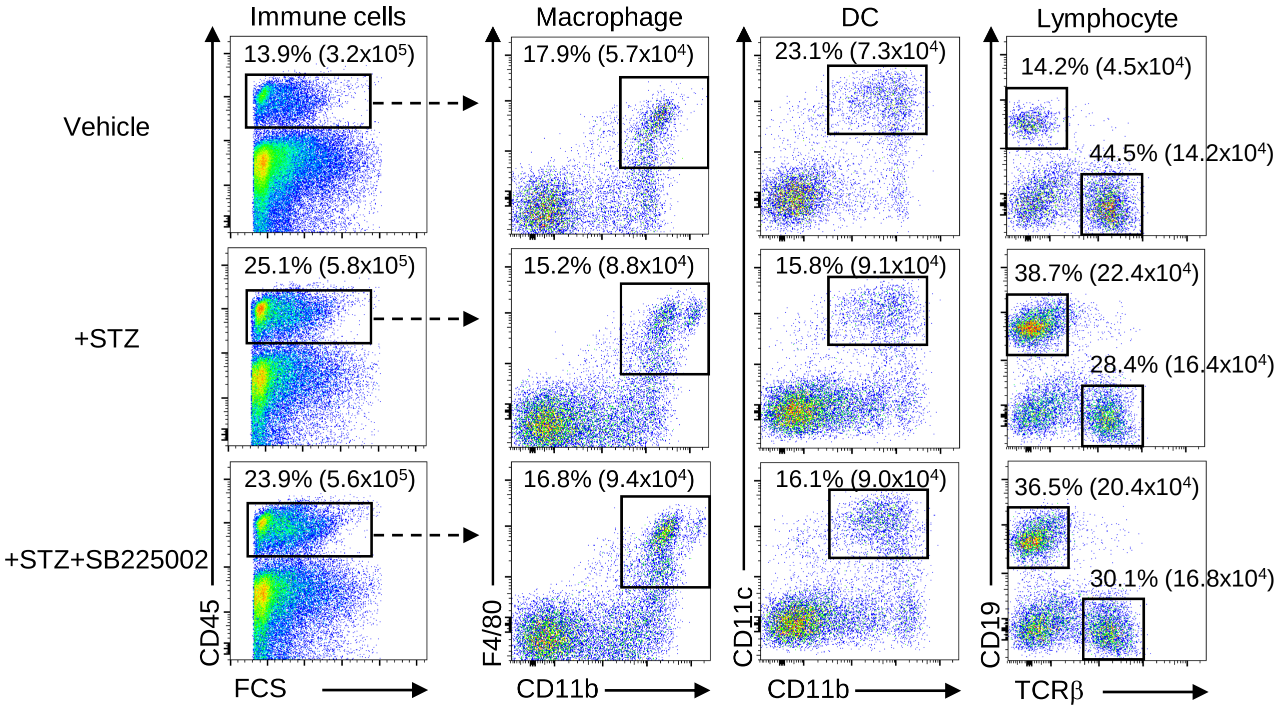

Figure S3. Population of infiltrating cells in the pancreatic islets of NOD mice after streptozotocin and SB225002 treatments. Pancreatic islet cells from 6-wk-old NOD mice were recovered 12h after STZ injection (i.p. 80mg/kg) and infiltrating cells were analyzed by flow cytometry. Percentage and absolute number (in brackets) of each population is represented. Data are representative of 2 independents experiments each with 2 pooled mice per group.
