# Supplementary material for: Macrophages and β-cells are responsible for CXCR2-mediated neutrophil infiltration of the pancreas during autoimmune diabetes
Source: EMBO Mol Med. 2014 Jun 26;6(8):1090–104. doi: 10.15252/emmm.201404144 (PMC4154135; doi:10.15252/emmm.201404144)
Supplement: Supplementary file 4 [file emmm0006-1090-sd4.pdf]

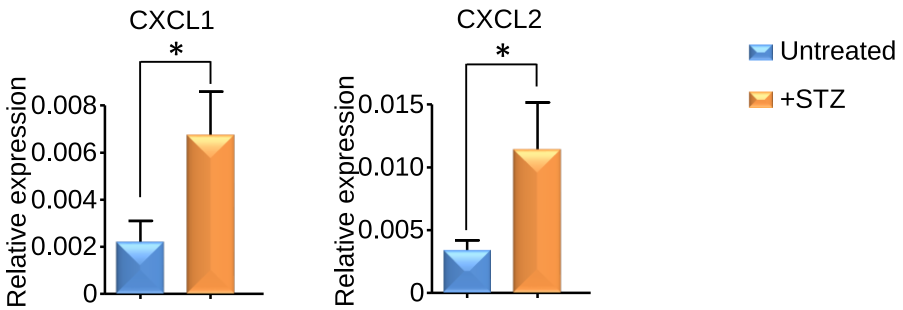

Figure S4. mRNA expression of chemokines in pancreatic islets treated by streptozotocin. Pancreatic islets pooled from independent NOD scid mice were handpicked and cultured overnight to ensure the release of potential infiltrating immune cells. Then islets were handpicked a second time and separated in two groups: one treated with the vehicle and the other one with streptozotocin (STZ, 0.5 mg/mL) . Both islet preparations were cultured for six additional hours and then recovered for analysis. The mRNA expression of chemokine genes were analyzed by quantitative-PCR. Data were normalized to gapdh housekeeping gene. Data are Data are median +/– interquartile range (scatter plot) from two independent experiments with two independent mice for each group. *: $P < 0.05$.
