# Supplementary material for: Macrophages and β-cells are responsible for CXCR2-mediated neutrophil infiltration of the pancreas during autoimmune diabetes
Source: EMBO Mol Med. 2014 Jun 26;6(8):1090–104. doi: 10.15252/emmm.201404144 (PMC4154135; doi:10.15252/emmm.201404144)
Supplement: Supplementary file 5 [file emmm0006-1090-sd5.pdf]

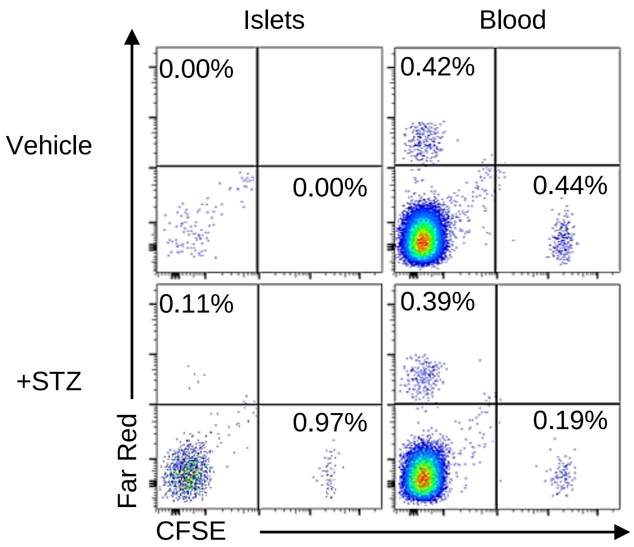

Figure S5. Neutrophil recruitment in the pancreas requires CXCR2 expression on the neutrophil. Neutrophils (CD45$^+$ CD11b$^+$ Ly6G$^+$) were facs-sorted from the BM of 6-wk-old NOD mice and cells were treated or not ex vivo with SB225002. Then treated and untreated neutrophils were stained with Cell trace® FarRed or CSFE, respectively and then cotransfer in 6-wk-old NOD mice. Twelve hours after transfer, mice were injected with streptozotocin and 12 h after, cells were harvested from the pancreatic islets and blood and stained for CD45, CD11b, and Ly6G expressions. Data represented the frequency of transferred neutrophils among the total neutrophil population. Data are representative of two independent experiments with three pooled mice for each group.
