# Supplementary material for: Macrophages and β-cells are responsible for CXCR2-mediated neutrophil infiltration of the pancreas during autoimmune diabetes
Source: EMBO Mol Med. 2014 Jun 26;6(8):1090–104. doi: 10.15252/emmm.201404144 (PMC4154135; doi:10.15252/emmm.201404144)
Supplement: Supplementary file 6 [file emmm0006-1090-sd6.pdf]

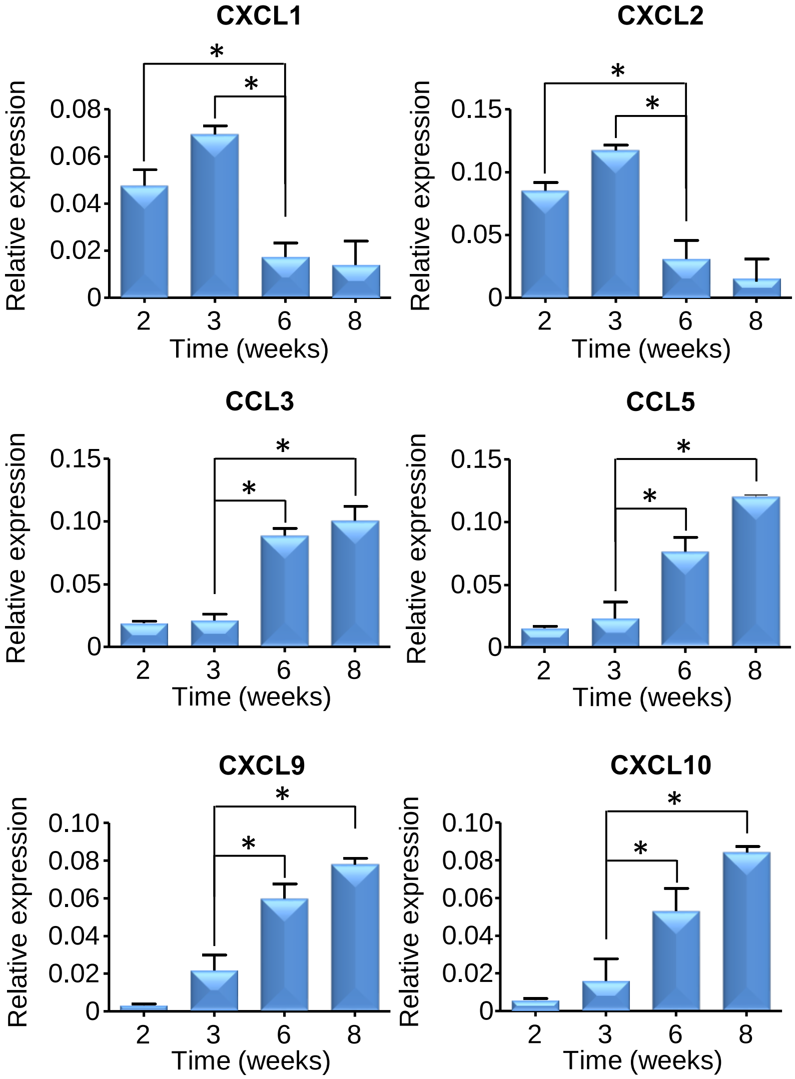

Figure S6. mRNA expression of CCR1 ligands in the pancreatic islets of NOD mice at various ages. Purified pancreatic islets were collected in RLT buffer with 1% of β-mercaptoethanol. mRNA was isolated using RNeasy Mini Kit (Qiagen) and reverse transcripted with Superscript III (Roche). Quantitative-PCR was performed with SYBR Green and analyzed on a LightCycler 480 for the expression of chemokine genes. Data were normalized to *gapdh* housekeeping gene. Data are mean values +/– SEM (scatter plot) from three independent experiments with four independent mice for each group. *: $P < 0.05$.
