# Supplementary material for: Macrophages and β-cells are responsible for CXCR2-mediated neutrophil infiltration of the pancreas during autoimmune diabetes
Source: EMBO Mol Med. 2014 Jun 26;6(8):1090–104. doi: 10.15252/emmm.201404144 (PMC4154135; doi:10.15252/emmm.201404144)
Supplement: Supplementary file 7 [file emmm0006-1090-sd7.pdf]

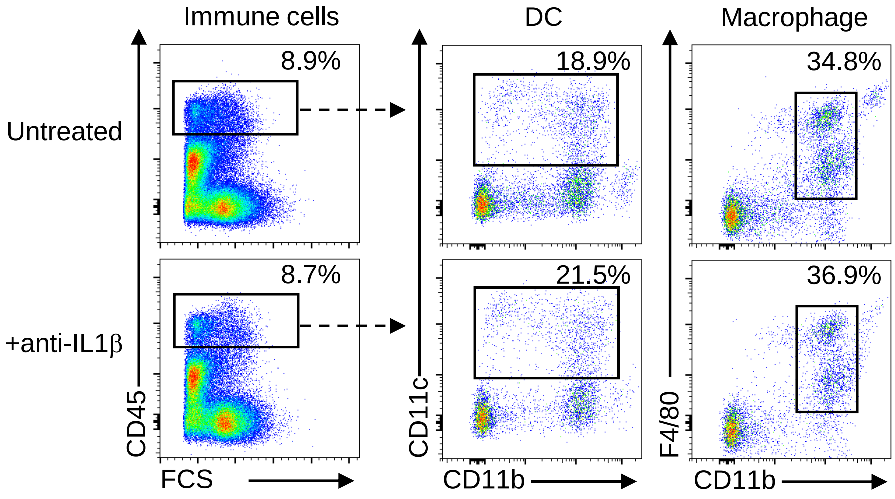

Figure S7. Population of infiltrating cells in the pancreatic islets of 3-wk-old NOD mice after anti-IL1β treatment. NOD mice were treated between 10 and 20 days of age (4 injections of anti-IL1β mAb, 200 µg/mouse/injection). Pancreatic islet cells were recovered and analyzed by flow cytometry. Percentage of each population is represented after gating on $CD45^+$ cells. Data are representative of 3 independents experiments.
