# Supplementary material for: Macrophages and β-cells are responsible for CXCR2-mediated neutrophil infiltration of the pancreas during autoimmune diabetes
Source: EMBO Mol Med. 2014 Jun 26;6(8):1090–104. doi: 10.15252/emmm.201404144 (PMC4154135; doi:10.15252/emmm.201404144)
Supplement: Supplementary file 8 [file emmm0006-1090-sd8.pdf]

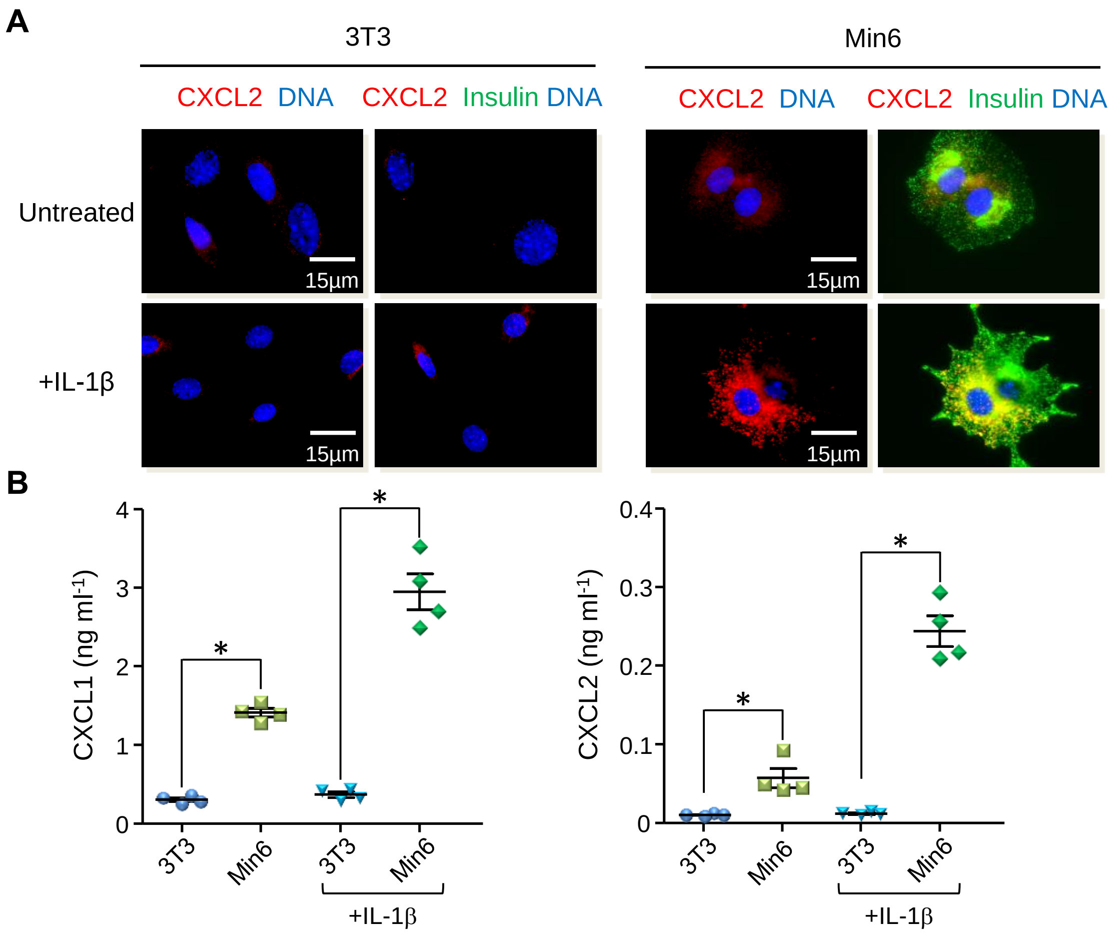

Figure S8. β-cells produce CXCL1 and CXCL2 under inflammatory conditions in vitro. (**a, b**) 3T3 fibroblasts and Min-6 β cells were cultured in absence or presence of rmIL-1β for 24h and then stained for the expression of CXCL2, insulin and DNA (**a**) and CXCL1 and CXCL2 productions were measured by ELISA in the culture supernatants (**b**). Data are mean values +/– SEM (scatter plot) (**b**) or are representative (**a**) from four independent experiments. *: P < 0.05 comparing group as indicated.
