# Supplementary material for: Macrophages and β-cells are responsible for CXCR2-mediated neutrophil infiltration of the pancreas during autoimmune diabetes
Source: EMBO Mol Med. 2014 Jun 26;6(8):1090–104. doi: 10.15252/emmm.201404144 (PMC4154135; doi:10.15252/emmm.201404144)
Supplement: Supplementary file 9 [file emmm0006-1090-sd9.pdf]

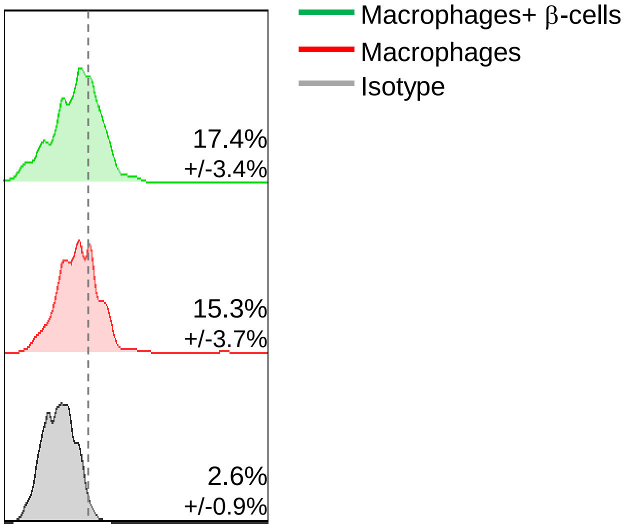

Figure S9. CXCL2 expression by macrophages after culture with Min-6 β-cells. Purified pancreatic macrophages from 3-wk-old NOD mice were cultured 48 h alone or with Min-6 β-cells in a Transwell system (macrophages in the down chamber and Min6 cells in the upper chamber) for 48h in the presence of Brefeldin A the last 4h. Then macrophages were stained for CD11b and F4/80 surface expressions and for CXCL2 intracellular expression. Data are mean values +/– SEM from two independent experiments each performed with two pools of two mice.
