# Supplementary material for: Macrophages and β-cells are responsible for CXCR2-mediated neutrophil infiltration of the pancreas during autoimmune diabetes
Source: EMBO Mol Med. 2014 Jun 26;6(8):1090–104. doi: 10.15252/emmm.201404144 (PMC4154135; doi:10.15252/emmm.201404144)
Supplement: Supplementary file 10 [file emmm0006-1090-sd10.pdf]

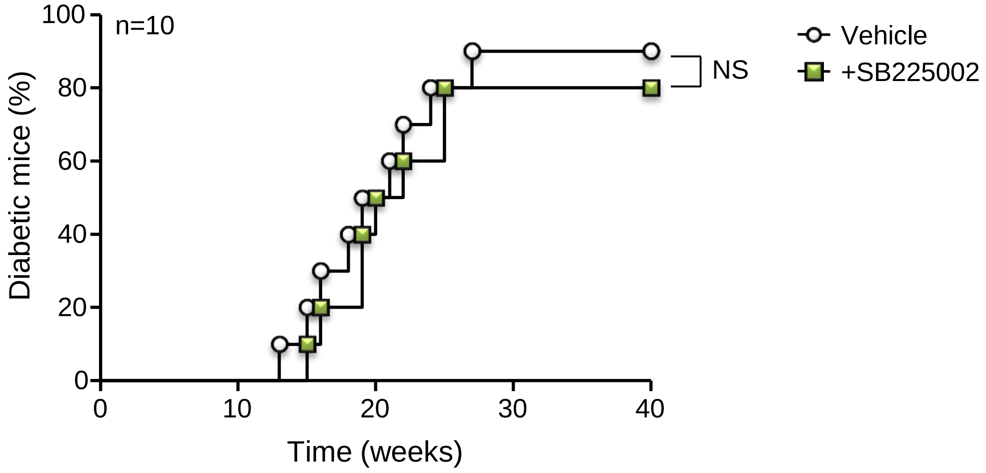

Figure S10. CXCR2 blockage in prediabetic NOD mice does not affect diabetes development. Female NOD mice were treated with SB225002 or vehicle between 8-10 weeks of age (4 injections) and incidence of diabetes was followed. NS: not significant difference, n=10 mice/group.
