# Supplementary material for: Macrophages and β-cells are responsible for CXCR2-mediated neutrophil infiltration of the pancreas during autoimmune diabetes
Source: EMBO Mol Med. 2014 Jun 26;6(8):1090–104. doi: 10.15252/emmm.201404144 (PMC4154135; doi:10.15252/emmm.201404144)
Supplement: Supplementary file 11 [file emmm0006-1090-sd11.pdf]

Table S1

Chemoattractant	Treatment	Median cell count +/- interquartile	n experiment
Medium	-	2 485 +/- 369	6
rmCXCL2 (10ng mL^{-1})	-	6 284 +/- 546	2
rmCXCL2 (100ng mL^{-1})	-	7 712 +/- 427	2
rmCXCL2 (1000ng mL^{-1})	-	10 125 +/- 920	6
rmCXCL2 (1000ng mL^{-1})	SB225002	2 224 +/- 684	6
3-wk-old NOD islet SUP	-	11 234 +/- 1 284	6
3-wk-old NOD islet SUP	SB225002	2 796 +/- 386	6
3-wk-old NOD islet SUP	aCXCL1	6 387 +/- 736	6
3-wk-old NOD islet SUP	aCXCL2	5 726 +/- 547	6
3-wk-old NOD islet SUP	aCXCL1+aCXCL2	2 932 +/- 429	6
3-wk-old NOD islet SUP	IgG	9 928 +/- 341	6
3-wk-old NOD islet SUP	WRW4	9 482 +/- 981	6
6-wk-old NOD islet SUP	-	4 916 +/- 1 268	4
3-wk-old C57BL/6 islet SUP	-	2 365 +/- 586	4
3-wk-old BALB/c islet SUP	-	2 472 +/- 750	4
αIL1β-treated 3-wk-old NOD islet SUP	-	4 983 +/- 1 965	4

Table S1. Absolute number of migrating neutrophils recovered in *in vitro* migration assays.

Migration assays were performed using 24-well microchemotaxis chambers and 5-µm PVP-free polycarbonate filter. 10e5 sorted blood-neutrophils were added to the upper compartment and 4h latter migrating cells were counted. Data are median +/– interquartile range from two to six independent experiments each performed in duplicate.
