# Supplementary material for: Macrophages and β-cells are responsible for CXCR2-mediated neutrophil infiltration of the pancreas during autoimmune diabetes
Source: EMBO Mol Med. 2014 Jun 26;6(8):1090–104. doi: 10.15252/emmm.201404144 (PMC4154135; doi:10.15252/emmm.201404144)
Supplement: Supplementary file 12 [file emmm0006-1090-sd12.pdf]

EMBO Molecular Medicine

Macrophages and -cells are responsible for CXCR2-mediated neutrophil infiltration of the pancreas during autoimmune diabetes

Julien Diana and Agnès Lehuen

Corresponding author: Julien DIANA, Inserm U1151; Necker-Enfants Malades Institute INEM ; University Paris-Descartes

---

Review timeline:

Submission date:	19 February 2014
Editorial Decision:	14 March 2014
Resubmission:	07 April 2014
Editorial Decision:	26 May 2014
Revision received:	27 May 2014
Accepted:	27 May 2014

Transaction Report:

(Note: With the exception of the correction of typographical or spelling errors that could be a source of ambiguity, letters and reports are not edited. The original formatting of letters and referee reports may not be reflected in this compilation.)

Editor: Céline Carret

1st Editorial Decision 14 March 2014

Thank you for the submission of your research manuscript to our editorial office. We have now received the enclosed reports on it. As you will see, referee 3 is rather positive about it. The other two reviewers however, while considering the results potentially interesting, raise serious concerns regarding the significance and conceptual advance of the data and pinpoint several technical issues that preclude a solid interpretation of the experimental evidence provided. These two reviewers call for better justified statistics, higher number of animal used per experiments, FACS numbers rather than percentages (we agree with referee 2 that a low number of cells would greatly impact on the significance of the observations), etc.

Given the low enthusiasm of two referees out of three and the amount of work likely to be required to address the significance issue, I am afraid that we do not feel it would be productive to call for a revised version of your manuscript at this stage and therefore we cannot offer to publish it.

Nevertheless, as the topic is interesting we would have no objection to consider a new manuscript on the same topic if at some time in the near future you obtained data that would considerably strengthen the message of the study and address the referees concerns in full. To be completely clear, however, I would like to stress that if you were to send a new manuscript this would be treated as a new submission rather than a revision and would be reviewed afresh, in particular with respect to the literature and the novelty of your findings at the time of resubmission. If you decide to follow this route, please make sure you nevertheless upload a letter of response to the referees' comments.

At this stage, though, I am sorry to have to disappoint you. I nevertheless hope, that the referee comments will be helpful in your continued work in this area and I thank you for considering EMBO Molecular Medicine.

***** Reviewer's comments *****

Referee #1 (Comments on Novelty/Model System):

The authors have built upon their original findings detailing neutrophil presence at early time points in the pancreas of NOD mice. In this current manuscript they examine the mechanisms underpinning neutrophil recruitment, in particular the role of CXCR2 and its ligands in the trafficking and recruitment of these cells to the pancreas. A role for macrophages and involvement of beta cells themselves in this process is also documented. IL-1beta produced by macrophages is suggested to increase CXCR2 ligand expression by beta cells. The study relies on the effects and specificity of the inhibitor SB225002 on neutrophils as well as clodronate liposomes to deplete macrophages- although of course the latter would deplete any phagocytic cell population.

The data is on the whole convincing but there are some concerns. There is a certain inclarity about the statistics used in the manuscript. In the Materials and Methods it is stated that a Mann Whitney non parametric test was used for data other than the NOD survival curves. This indeed would be the appropriate test In some cases only 4 samples per group (1A, 2C, 3A and E) and often they arise from 2 independent experiments with 2 mice in each group- the numbers are on the small side experimentally and ideally larger sample sizes should be compared.Furthermore a paired t test is used in Fig 4- it was unclear why this was used on this occasion.

The figure legend in Fig E4 is rather ambiguous. It seems from this legend as though the controls and the STZ treatment groups were done on different days. Did they mean that the islets were initially handpicked and left in culture for 24 hours before dividing into 2 groups one of which remained in culture medium alone and the other had added STZ and the 2 groups were cultured for a further 6 hours before analysis. This figure legend has to be made more explicit.

The numbers of mice used in E10 should be included in the figure legend.

The authors describe analysing insulin+CD45- cells but do not provide the details of their preparation for FACS analysis in the Materials and Methods- information is only given for cytology and presumbaly there were differences to obtain the cells for good FACs profiling.

Referee #1 (Remarks):

This is an interesting manuscript which provides data which extends their original findings detailing neutrophil presence at early time points in the pancreas of NOD mice. In this current manuscript they examine the mechanisms underpinning neutrophil recruitment, in particular the role of CXCR2 and its ligands in the trafficking and recruitment of these cells to the pancreas. A role for macrophages and involvement of beta cells themselves in this process is also documented. IL-1beta produced by macrophages is suggested to increase CXCR2 ligand expression by beta cells. The study relies on the effects and specificity of the inhibitor SB225002 on neutrophils as well as clodronate liposomes to deplete macrophages- although of course the latter would deplete any phagocytic cell population.

The data is on the whole convincing but there are some concerns. There is a certain inclarity about the statistics used in the manuscript. In the Materials and Methods it is stated that a Mann Whitney non parametric test was used for data other than the NOD survival curves. A paired t test is used in Fig 4- it was unclear why this was used on this occasion at any rate it should have been also included in the Materials and Methods. The Mann Whitney is indeed would the appropriate test for much of the data sets collected in this paper. However, the data sets are in some cases rather small- only 4 samples per group (1A, 2C, 3A and E) and often they arise from 2 independent experiments

with 2 mice in each group. These numbers are on the small side experimentally and ideally larger sample sizes should be compared- although the differences are mainly very clear.

The figure legend in Fig E4 is rather ambiguous. It seems from this legend as though the controls and the STZ treatment groups were done on different days. Did they mean that the islets were initially handpicked and left in culture for 24 hours before dividing into 2 groups one of which remained in culture medium alone and the other had added STZ and the 2 groups were cultured for a further 6 hours before analysis. This figure legend has to be made more explicit.

The numbers of mice used in E10 should be included in the figure legend.

The authors describe analysing insulin+CD45- cells but do not provide the details of their preparation for FACS analysis in the Materials and Methods- information is only given for cytology and presumbaly there were differences to obtain the cells for good FACs profiling.

Referee #2 (Remarks):

This is an interesting study that focuses on early events in NOD mice, seemingly before the adaptive immune system becomes activated and islet autoantigen-specific T cells invade the pancreas. The study follows other recent work by these authors on the presence of neutrophils in the pancreatic islets, and from that perspective is to an extent incremental.

The key findings are that, alongside an influx of macrophages greater than that seen in a control strain, there is also an influx of neutrophils. This is transient and appears to peak at 3 weeks of age and is accompanied by a drop in neutrophils elsewhere (not followed up but implied to be the inverse of what is happening in tissues).

At 3 weeks of age the histological abnormalities in the NOD pancreas are very subtle. The FACS profiles in Figure 1A show the percentage of neutrophils among CD45 positive cells. This is a very confusing profile in several ways. First % of what is confusing - because there are many lymphocytes (in the left lower quadrant) in the spleen and blood but fewer in the islet profile. So somehow the neutrophils are being overemphasized. To me there are many macrophages and a small number of neutrophils in the islet profile shown - eg if you expressed the percentage as neutrophils/macrophages x 100 you would send a very different message. Also the absolute number of cells should be shown - not just the percentages. The profiles look "clean" as a result of gating on CD45 but in reality there must be very few cells present.

The drug SB225002 which is a chemokine antagonist was used to inhibit this infiltration of neutrophils. Chemokine induction was localized to both beta cells and macrophages. And appeared to be dependent on IL-1 production.

The main question is how does the neutrophil infiltration contribute to the development of diabetes as opposed to being an incidental finding perhaps somehow coupled to macrophage infiltration. A second question is whether adaptive immune responses are needed for myeloid cell infiltration or whether they are responsible for later macrophage infiltration?

Despite the interest in the data the impact of the findings is not sheeted home by a highly compelling in vivo experiment. IL-1 is seen as responsible for the infiltration but IL-1 antagonists do not prevent diabetes in NOD mice. Even the chemokine antagonist produces only a part effect in vivo, bearing in mind it was administered for a relatively short time.

Experiments I would be interested in seeing:

Does neutrophil infiltration occur in T cell deficient mice eg NOD SCID or Rag knockouts, or in mice in which a T-cell intervention protects from diabetes.

What is the role of the adaptive immune system in production of chemokines by beta cells?

In Figure 4A there is expression of CXCL2 also in B6 mice. Is there some way of quantitatively describing this to determine how different from NOD it is?

Referee #3 (Remarks):

Diana and Lehuen report that neutrophils are recruited in a CXCR2 dependent way to the islet of Langerhans and that macrophages and beta-cells play an important role in this process. The authors used the NOD model for type 1 diabetes and could reproduce their data of an early infiltration of the islets by neutrophils. Here they further demonstrate that the recruitment is largely CXCR2 dependent since blockade of both CXCR2 as well as CXCL1 and CXCL2 significantly reduced islet infiltration by neutrophils. Beta-cells and macrophages have been identified as the source for CXCL2. In addition the authors identified IL1beta produced by macrophages as main inducer of CXCL2 production. Macrophage depletion experiments and IL-1beta blockade confirmed these findings.

This is an excellent article that has the clear message that the CXCL1/2-CXCR2 pathway is important for the initial steps in the pathogenesis of T1D in the NOD mouse. The data are clear cut and the experiments have been executed carefully. The authors performed a multitude of control experiment to exclude experimental artifacts (displayed in several supplemental figures). The conclusions made by the authors are confirmed by the provided data.

Additional comments:

Figure 4: Although the immunohistologic signal of CXCL2 is not that convincing, together with the immunocytology of isolated beta-cells and the FACS data, it seems nevertheless clear that CXCL2 is produced by beta-cells and macrophages. It is unfortunate that no antibodies to CXCL1 are available. The authors could try to demonstrate CXCL1 production in isolated beta-cells, isolated whole islets or in beta cell lines on the RNA level by RT-PCR.

Figure 7B: A clear difference in islet infiltration is visible, but the blue color is somewhat overwhelming. I am sure the authors have some better pictures.

Page 9: paragraph title: CXCR2 (C missing)

Resubmission 07 April 2014

Manuscript EMM-2014-03990

Macrophages and β-cells are responsible for CXCR2-mediated neutrophil infiltration of the pancreas during autoimmune diabetes; by Diana et al.

Point by point to the reviewer's comments:

First of all we would like to thank the editor and the reviewers for their positive comments and their encouraging and constructive criticisms of our manuscript. According to their commentaries we have addressed the issues raised by the reviewers as detailed above.

Reviewer: 1

Comments on Novelty/Model System:

The authors have built upon their original findings detailing neutrophil presence at early time points in the pancreas of NOD mice. In this current manuscript they examine the mechanisms underpinning neutrophil recruitment, in particular the role of CXCR2 and its ligands in the trafficking and recruitment of these cells to the pancreas. A role for macrophages and involvement of beta cells themselves in this process is also documented. IL-1beta produced by macrophages is suggested to increase CXCR2 ligand expression by beta cells. The study relies on the effects and specificity of the inhibitor SB225002 on neutrophils as well as clodronate liposomes to deplete macrophages- although of course the latter would deplete any phagocytic cell population.

The data is on the whole convincing but there are some concerns. There is a certain inclarity about the statistics used in the manuscript. In the Materials and Methods it is stated that a Mann Whitney non parametric test was used for data other than the NOD survival curves. This indeed would be the appropriate test In some cases only 4 samples per group (1A, 2C, 3A and E) and often they arise from 2 independent experiments with 2 mice in each group- the numbers are on the small side experimentally and ideally larger sample sizes should be compared. Furthermore a paired t test is used in Fig 4- it was unclear why this was used on this occasion.

We have chosen to use the non-parametric Mann Whitney U-Test to analyze our data (except for diabetes incidence experiments) since we believe that this statistical test is the most appropriate. Indeed most of the other tests requires that the distribution of variables shows a normal distribution. This condition cannot be reached if there are less than 20 independent values per group. The Mann-Whitney U-test can be used in our case as it can be applied for small samples (minimum 3 values are required). Moreover as requested by the reviewer we have performed additional experiments to increase the sample size in the figures 1A, 2C, 3A, 3E, 6A and 6B strengthening our conclusions. In the revised version of the manuscript we now provide 6 independent experiments for the figure 1A with 3 pooled mice per group (n=6), 8 independent experiments for the figure 2C with 4 pooled mice per group (n=8), 3 independent experiments for the figure 3A with 2 independent mice per group (n=6), 5 independent experiments for the figure 3E, 3 independent experiments for the figure 6A, B with 2 independent mice per group (n=6).

Yet we would like to mention that for many experiments each dot represents a pool of 3 or 4 mice increasing the weight of the data.

Regarding the figure 4C, we had chosen to use the pair t test since mean values +/-SEM were shown in the graph while Mann Whitney test compares median values. However to be homogenous, in the revised version of our manuscript we now show and analyze the median values using the Mann Whitney test.

The figure legend in Fig E4 is rather ambiguous. It seems from this legend as though the controls and the STZ treatment groups were done on different days. Did they mean that the islets were initially handpicked and left in culture for 24 hours before dividing into 2 groups one of which remained in

culture medium alone and the other had added STZ and the 2 groups were cultured for a further 6 hours before analysis. This figure legend has to be made more explicit.
We agree with the reviewer that the legend of the figure E4 has to be clarified. To be clear, control and STZ groups were done on the same day. First, pancreatic islets were handpicked from independent NOD scid mice and then pooled and cultured overnight to ensure the release of potential infiltrating immune cells (note: few immune cells were present in these islets since we used the NOD scid mice). The second day, islets were handpicked a second time and separated in two groups: one treated with the vehicle and the other one with STZ. Both islet preparations were cultured for six additional hours and then recovered for analysis. As requested by the reviewer the figure legend E4 has been revised accordingly.

The numbers of mice used in E10 should be included in the figure legend.
We agree with the reviewer remark that this information must be included, however the number of mice used in this experiment was already indicated in the first version of the manuscript. Ten mice per group were used in this experiment (supplementary Figure E10).

The authors describe analysing insulin+CD45- cells but do not provide the details of their preparation for FACS analysis in the Materials and Methods- information is only given for cytology and presumbaly there were differences to obtain the cells for good FACs profiling.
As requested by the reviewer we now better describe in the revised Materials and Methods (p14, l8) the protocol to analyze insulin$^+$ CD45$^-$ cells in the pancreatic islets by flow cytometry. There is no major difference in the way to prepare these cells for flow cytometry or cytology and antibodies used were working in both situations. However it is important to note that a specific setting of the flow cytometer (specific FCS and SSC parameters) is required to properly analyze the pancreatic beta cells due to their high granulometry and autofluorescence.

Reviewer: 2

This is an interesting study that focuses on early events in NOD mice, seemingly before the adaptive immune system becomes activated and islet autoantigen-specific T cells invade the pancreas. The study follows other recent work by these authors on the presence of neutrophils in the pancreatic islets, and from that perspective is to an extent incremental.

The key findings are that, alongside an influx of macrophages greater than that seen in a control strain, there is also an influx of neutrophils. This is transient and appears to peak at 3 weeks of age and is accompanied by a drop in neutrophils elsewhere (not followed up but implied to be the inverse of what is happening in tissues).

At 3 weeks of age the histological abnormalities in the NOD pancreas are very subtle. The FACS profiles in Figure 1A show the percentage of neutrophils among CD45 positive cells. This is a very confusing profile in several ways. First % of what is confusing - because there are many lymphocytes (in the left lower quadrant) in the spleen and blood but fewer in the islet profile. So somehow the neutrophils are being overemphasized. To me there are many macrophages and a small number of neutrophils in the islet profile shown - eg if you expressed the percentage as neutrophils/macrophages x 100 you would send a very different message. Also the absolute number of cells should be shown - not just the percentages. The profiles look "clean" as a result of gating on CD45 but in reality there must be very few cells present.

We perform our flow cytometry analysis of infiltrating pancreatic cells by gating on $CD45^+$ cells as classically described (Poirot et al. *PNAS* 2004; Ochi et al. *J. Exp. Med.* 2012; Pylayeva-Gupta et al. *Cancer Cell* 2012; Fu et al *Nature Immunology* 2012; Yin et al. *J. Immunol.* 2012; Fakhari et al. *J. Applied Biomed.* 2013). Indeed this strategy allows to focus on the cells of interest since in the pancreatic islets from 3-wk-old NOD mice the absolute number of immune cells is largely lower than the absolute number of non-immune endocrine cells. We agree with the reviewer remark that it is necessary to show the absolute number of immune cells present in the pancreatic islets, however we already showed these numbers in the first version of our manuscript (supplementary Figure E1A). We observed a significant increased number of neutrophils in the pancreatic islets of NOD mice at 3 weeks of age while neutrophils were undetectable in the islets from C57BL/6 or BALB/c mice. These data confirmed our previous data revealing by flow cytometry and immunohistology the presence of neutrophils specifically in the pancreatic islets of young NOD mice and not in prediabetic NOD mice or young C57BL/6 or BALB/c mice (Diana et al. *Nat. Med.* 2013).

Moreover we would like to stress that even if the absolute number of a cell type in the infiltrated islets is small it does not mean that this cell type does not play an important role in the disease. Indeed as clearly shown in our previous study (Diana et al. *Nat. Med.* 2013) neutrophils are required for the initiation of autoimmune diabetes in the NOD mice. The specific depletion of these cell type between 1 and 3 weeks of age and not latter (data not shown) strongly reduced the incidence of diabetes in the NOD mice. We had further showed that neutrophils were strongly activated in the pancreatic islets, released the CRAMP molecule and with the help of B1a cells stimulated plasmacytoid dendritic cells to secrete type I interferon (Summarize in Creusot *Nature Medicine* 2013). As for neutrophils, while plasmacytoid dendritic cells represent few cells into the pancreatic islets, they play a crucial role in the development of the disease through the secretion of high amount of type 1 interferon as revealed by depleting and blocking experiments.

Similarly, conventional dendritic cells are also critical for the initiation of the adaptive diabetogenic response by capturing beta cell antigens, carrying them to the draining lymph nodes where they present the peptides to the autoreactive T cells (Ganguly et al *Nature Rev. Immunol.* 2013). This critical course is however guarantee by a small number of conventional dendritic cells in the pancreatic islets (Yin et al *J. Immunol.* 2012). Likewise, we and others have described the protective role of invariant natural killer T (iNKT) cells against autoimmune diabetes despite the fact that these cells are present in very small number in the islets (Sharif et al. *Nature Medicine* 2001; Beaudoin et al. *Immunity* 2002; Diana et al. *J. Exp. Med.* 2011; Van Kaer *Nat. Rev. Immunol.* 2004). Finally, it has been extensively studied and demonstrated that peripheral tolerance in NOD mice is dependent on the balance of

effector and regulatory T cells more than by the absolute number of T cells in the pancreatic islets (Bour-Jordan et al. *JCI* 2004; Jeker et al. *Cold Spring Harbor Persp. Med.* 2012)

The drug SB225002 which is a chemokine antagonist was used to inhibit this infiltration of neutrophils. Chemokine induction was localized to both beta cells and macrophages. And appeared to be dependent on IL-1 production. The main question is how does the neutrophil infiltration contribute to the development of diabetes as opposed to being an incidental finding perhaps somehow coupled to macrophage infiltration.

It is important to keep in mind that in our previous publication (Diana et al. *Nat. Med.* 2013) we have demonstrated using specific depleting antibody the requirement of neutrophils for the initiation of diabetes in the NOD mice. In this previous study we further deciphered how neutrophils act in the pancreatic islets *via* the activation of plasmacytoid dendritic cells and the release of type I interferon. So, it is unlikely that neutrophils were incidentally recruited in the pancreatic islets without playing any role in the development of the disease. Our findings are also supported by two others studies revealing first the presence of neutrophils in the pancreas of recent-onset T1D patients and not in T2D patients or healthy individuals (Valle et al. *Diabetes* 2013); and second the deleterious role of neutrophils for the survival of grafted islets both in mouse diabetes model and T1D patients (Citro et al. *JCI* 2012). However this does not exclude that many other cell types (including macrophages) are required for the development of T1D as shown by us and many others (Lehuen et al. *Nat. Rev. Immunol.* 2010; Atkinson *Lancet* 2014). We also show in the present manuscript that macrophages are required for the recruitment of neutrophils into the islets by producing chemokines and by stimulating chemokine production by the beta cells. It has been described that macrophages are the first cells to infiltrate the islets during diabetes development (Dahlen et al. *J. Immunol.* 1998). Indeed, during the first postnatal weeks, waves of physiological β-cell death occur in rodents (Mathis et al. *Nature* 2001), pigs (Bock et al. *J Endocrinol.* 2003) and humans (Kassem et al. *Diabetes* 2000) likely due to organogenesis. This leads to the recruitment of macrophages required to eliminate cell debris. However, specifically in the NOD genetic background, these macrophages produce exaggerated amounts of inflammatory cytokines leading to a persistent inflammation of the pancreas (Stoffels et al. *J. Autoimmun.* 2004). Remarkably, this genetic defect of macrophages has been also identified in human with monocytes isolated from the blood of T1D patients (Bradshaw et al. *J. Immunol.* 2009). This peculiar phenotype of "autoimmune"-macrophages may be responsible for a state of inflammation or stress in the pancreatic islets, leading to the recruitment of other innate immune cells to assist the struggling macrophages.

A second question is whether adaptive immune responses are needed for myeloid cell infiltration or whether they are responsible for later macrophage infiltration?

In our previous study, we showed the kinetic of the various cell types infiltrating the pancreatic islets of NOD mice from 2 to 6 weeks of age (Diana et al. *Nat. Med.* 2013). We observed that while dendritic cells and neutrophils were present at 2-3 weeks of age, T cells were not detected at this age but started to infiltrate the pancreatic islets at 4-6 weeks of age. Consequently it is rather unlikely that the recruitment of myeloid cells in the pancreatic islets at 2-3 weeks of age may be depend on T cells not yet present in the islets. Some B cells were present in the islets at early ages however we showed that they are innate-like B1a B cells (Diana et al. *Nat. Med.* 2013). Accordingly, macrophages and dendritic cells are the first cell types to infiltrate the pancreatic islets of NOD mice during diabetes development and this recruitment was shown to be independent of T cells (Dahlen et al. *J. Immunol.* 1998).

Despite the interest in the data the impact of the findings is not sheeted home by a highly compelling in vivo experiment. IL-1 is seen as responsible for the infiltration but IL-1 antagonists do not prevent diabetes in NOD mice. Even the chemokine antagonist produces only a part effect in vivo, bearing in mind it was administered for a relatively short time.

We agree with the reviewer that the role of IL-1b in the development of T1D remains to be fully elucidated since the first observation that IL-1b plays a deleterious role in the pathogenesis of T1D (Mandrup-Poulsen et al *Diabetologia* 1996). IL-1b has a pleiotropic role in the pancreatic islets, being

directly toxic for beta cells (Böni-Schnetzler et al. *Endocrinology* 2009) and stimulating effector cell survival while reducing the regulatory compartment (O'Sullivan et al. *J. Immunol.* 2006). Consequently, It was shown that IL-1 antagonists provide some protective effect against T1D (Cailleau et al. *Diabetes* 1997; Nicoletti et al. *Eur. J. Immunol.* 1994; Thomas et al. *Diabetes* 2004). A recent study also shows a synergistic reversal of diabetes in NOD mice with anti-CD3 and interleukin-1 blockade (Ablamunits et al *Diabetes* 2012). Another recent study revealed that IL-1 antagonism reduces hyperglycemia and tissue inflammation in type 2 diabetic rat (Ehses et al. *PNAS* 2009). Our personal data show that IL-1b neutralization in NOD mice between 1 and 3 weeks of age reduces significantly the development of the disease (see below, Figure R1). However, as observed for many cell types and cytokines in the context of autoimmune diabetes, IL-1b is probably not the unique cytokine responsible for the initiation of the disease and in its absence other cytokines (i.e. TNF, IFNγ, IFNα…) may exert some compensatory effects.

Experiments I would be interested in seeing:

Does neutrophil infiltration occur in T cell deficient mice eg NOD SCID or Rag knockouts, or in mice in which a T-cell intervention protects from diabetes.

This interesting experiment has been performed using NOD scid mice in our previous study (Figure S15 from Diana et al. *Nat. Med.* 2013, see below). We showed that in absence of T cells, neutrophils remained recruited in the pancreatic islets of 3-wk-old NOD scid mice. However according to the role of B1a cells in the activation of pancreatic neutrophils, in absence of B cells pancreatic neutrophils were not activated and did not express CRAMP at their surface. These data demonstrate that lymphoid cells are not necessary for the recruitment of the neutrophils into the pancreatic islets of young NOD mice but are required for their activation.

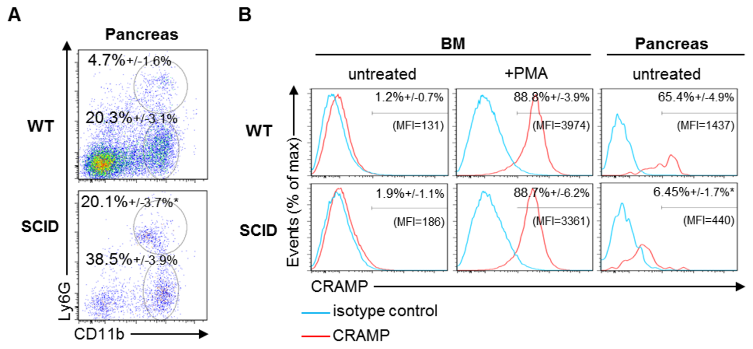

Figure S15. The activation but not the recruitment of neutrophils are affected in the pancreas of NOD SCID mice. (**a**) Cells were recovered from pancreatic islets of 3-week-old NOD mice and stained with anti-CD45, anti-CD11b and anti-Ly6G mAbs. Data represent the frequency of cells among CD45$^+$ cells. (**b**) Cells were recovered from bone marrow (BM) or pancreatic islets from NOD mice and stained with anti-CD45, anti-CD11b and anti-Ly6G mAbs, and then with anti-CRAMP pAb and anti-rabbit mAb. In some conditions cells were stimulated for 20 min with PMA (50nM) before staining. Data are mean values +/- s.e.m. of three independent experiments with four pooled mice for each group. *: P < 0.05 for SCID group compared to WT group. *(From Diana et al. Nat Med 2013)*

What is the role of the adaptive immune system in production of chemokines by beta cells?
As shown in the figure E4 of the present manuscript, islets from NOD scid mice produced chemokines after streptozotocin treatment excluding a role for lymphoid cells in this process. Moreover we show in this study and it has been shown by other groups that beta cells produce many chemokines in response to IL-1b, cytokine produced by myeloid and not lymphoid cells (Eizirik et al. *Nat Rev Endocrinol* 2009). Indeed beta cells are particularly sensitive to IL-1b since they expression IL-1R more than any other cell type in mammals (Scarim et al. *Biochim. Biophys. Acta.* 1997). Consequently, we believe that the adaptive immune system do not influence the production of chemokines by beta cells. Accordingly it has been shown that autoreactive T cells are recruited into the pancreatic islets subsequently to the production of chemokines by the beta cells (Frigerio et al *Nature* 2002).

In Figure 4A there is expression of CXCL2 also in B6 mice. Is there some way of quantitatively describing this to determine how different from NOD it is?
We agree with the reviewer that it is critical to quantify CXCL1/2 production in islets from NOD and C57BL/6 mice. However we already showed this experiment in the previous version of our manuscript (Figure 3A-B). By ELISA, we showed that 3-wk-old NOD-islets spontaneously secrete significantly higher amount of CXCL1 and CXCL2 than 3-wk-old C57BL/6-islets or 3-wk-old BALB/c-islets. Accordingly, the supernatants from C57BL/6- or BALB/c-islet cultures failed to recruit neutrophils *in vitro* contrary to supernatants from NOD-islet cultures (Figure 3E).

Reviewer: 3

Diana and Lehuen report that neutrophils are recruited in a CXCR2 dependent way to the islet of Langerhans and that macrophages and beta-cells play an important role in this process. The authors used the NOD model for type 1 diabetes and could reproduce their data of an early infiltration of the islets by neutrophils. Here they further demonstrate that the recruitment is largely CXCR2 dependent since blockade of both CXCR2 as well as CXCL1 and CXCL2 significantly reduced islet infiltration by neutrophils. Beta-cells and macrophages have been identified as the source for CXCL2. In addition the authors identified IL1beta produced by macrophages as main inducer of CXCL2 production. Macrophage depletion experiments and IL-1beta blockade confirmed these findings.

This is an excellent article that has the clear message that the CXCL1/2-CXCR2 pathway is important for the initial steps in the pathogenesis of T1D in the NOD mouse. The data are clear cut and the experiments have been executed carefully. The authors performed a multitude of control experiment to exclude experimental artifacts (displayed in several supplemental figures). The conclusions made by the authors are confirmed by the provided data.

Additional comments:

Figure 4: Although the immunohistologic signal of CXCL2 is not that convincing, together with the immunocytology of isolated beta-cells and the FACS data, it seems nevertheless clear that CXCL2 is produced by beta-cells and macrophages. It is unfortunate that no antibodies to CXCL1 are available. The authors could try to demonstrate CXCL1 production in isolated beta-cells, isolated whole islets or in beta cell lines on the RNA level by RT-PCR.

We already showed in the first version of our manuscript the mRNA expression of CXCL1 in whole islets from NOD mice. As for CXCL2 this expression was significantly higher in 2-3 wks old NOD mice compared to 6-8 wks old NOD mice (Figure E6). Anyway we have attempted to set up again CXCL1 staining for flow cytometry. We now show that, as for CXCL2, CXCL1 is express exclusively by insulin^{+} beta cells and macrophages in the pancreatic islets of 3-wk-old NOD mice (new figure 4C). We conclude that the two high affinity ligands for CXCR2 were produced at the protein level by both macrophages and beta cells in the pancreatic islets of young NOD mice.

Figure 7B: A clear difference in islet infiltration is visible, but the blue color is somewhat overwhelming. I am sure the authors have some better pictures.

First we would like to apologize for the low quality of the pictures shown in the first version of the manuscript. We now provide more satisfactory pictures showing the difference in islet infiltration between vehicle- or SB225002-treated NOD mice (new figure 7B).

Page 9: paragraph title: CXCR2 (C missing)

We apologize for this mistake and we have modified this paragraph title accordingly (p9, l20).

2nd Editorial Decision 26 May 2014

Thank you for the submission of your revised manuscript to EMBO Molecular Medicine. I sincerely apologise for the long delay in getting back to you. We have now received the enclosed reports from the referees that were asked to re-assess it. As you will see the reviewers are now globally supportive and I am pleased to inform you that we will be able to accept your manuscript pending final editorial amendments.

Please submit your revised manuscript as soon as possible but within 2 weeks.

I look forward to seeing a new revised version of your article.

***** Reviewer's comments *****

Referee #1 (Remarks):

In my view there are still major questions over the validity, significance and reproducibility of the work in this study - but time will tell.

Referee #2 (Comments on Novelty/Model System):

This is an apporpriate model system to study the early events in T!D. The authors have answered all the questions raised by this reviewer previously and have altered the manuscript accordingly to this reviewer's satisfaction.

Referee #2 (Remarks):

The authors have addressed the questions raised by this reviewer and amended their manuscript accordingly.

© EMBO
